# The IL-1RI Co-Receptor TILRR (*FREM1* Isoform 2) Controls Aberrant Inflammatory Responses and Development of Vascular Disease

**DOI:** 10.1016/j.jacbts.2017.03.014

**Published:** 2017-08-28

**Authors:** Sarah A. Smith, Andriy O. Samokhin, Mabruka Alfaidi, Emer C. Murphy, David Rhodes, W. Mike L. Holcombe, Endre Kiss-Toth, Robert F. Storey, Siu-Pok Yee, Sheila E. Francis, Eva E. Qwarnstrom

**Affiliations:** a Department of Infection, Immunity & Cardiovascular Disease, University of Sheffield, Sheffield, United Kingdom; b Department of Computer Science, University of Sheffield, Sheffield, United Kingdom; c Department of Cell Biology, University of Connecticut, Farmington, Connecticut; d Center for Mouse Genome Modification, University of Connecticut, Farmington, Connecticut

**Keywords:** heparan sulfate proteoglycan, interleukin-1 receptor, IL-1RI, NF-κB, TILRR, ApoE, apolipoprotein E, DK, double knockout, GAPDH, glyceraldehyde 3-phosphate dehydrogenase, iBALT, inducible bronchus-associated lymphoid tissue, IgG, immunoglobulin G, IκBα, inhibitor kappa B alpha, IL, interleukin, IL-1RI, interleukin-1 receptor type I, KO, knockout, LDLR^–/–^, low-density lipoprotein receptor^–/–^, LPS, lipopolysaccharide, NF-κB, nuclear factor-kappa B, NSTEMI, non–ST-segment elevation myocardial infarction, PBS, phosphate-buffered saline, PCR, polymerase chain reaction, qPCR, quantitative polymerase chain reaction, SDS, sodium dodecyl sulfate, STEMI, ST-segment elevation myocardial infarction, TILRR, toll-like and interleukin-1 receptor regulator

## Abstract

Expression of the interleukin-1 receptor type I (IL-1RI) co-receptor Toll-like and interleukin-1 receptor regulator (TILRR) is significantly increased in blood monocytes following myocardial infarction and in the atherosclerotic plaque, whereas levels in healthy tissue are low. TILRR association with IL-1RI at these sites causes aberrant activation of inflammatory genes, which underlie progression of cardiovascular disease. The authors show that genetic deletion of TILRR or antibody blocking of TILRR function reduces development of atherosclerotic plaques. Lesions exhibit decreased levels of monocytes, with increases in collagen and smooth muscle cells, characteristic features of stable plaques. The results suggest that TILRR may constitute a rational target for site- and signal-specific inhibition of vascular disease.

## Highlights

• The IL-1RI co-receptor, TILRR, is a potent amplifier of IL-1–induced responses.
• Blocking TILRR inhibits IL-1 receptor function and activation of inflammatory genes.
• TILRR expression is high in atherosclerotic lesions but low in healthy tissue, allowing distinct inhibition at sites of inflammation.
• Genetic deletion of TILRR and antibody blocking of TILRR function reduce plaque development and progression of atherosclerosis. Lesions exhibit low levels of macrophages and increased levels of smooth muscle cells and collagen, characteristics of stable plaques.

## Visual Abstract

**Figure:**
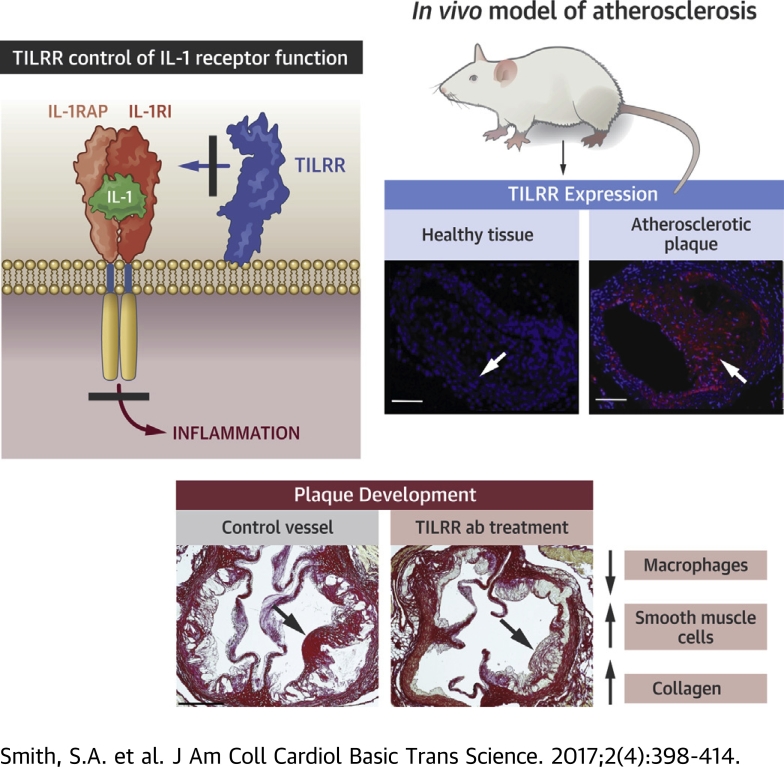

Receptors of the toll-like and interleukin (IL)-1 family are central to control of immunity and inflammation, and their importance in disease is well documented 1, 2, 3, 4. Activation is induced by ligand binding and by association of system-specific co-receptors, which regulate signal amplification and transcriptional activity 1, 4, 5. Changes in co-receptor expression and causative mutations impact responses to infection, tissue damage and stress, and affect development of disease (5).

Proteoglycans and glycosylated proteins act as co-receptors in a number of regulatory systems. Recruited to the receptor complex, they control receptor function, ligand binding, and extracellular interactions associated with aberrant signal activation and disease 6, 7, 8. The IL-1 receptor type I (IL-1RI), and its ligand, the cytokine IL-1, are potent activators of nuclear factor-kappa B (NF-κB) and intrinsically linked with acute and chronic inflammation (9). Dysregulation of NF-κB and IL-1-induced gene activity underlie development and progression of conditions such as atherosclerosis 10, 11. Our earlier studies identified Toll-like and IL-1 receptor regulator (TILRR) (*FREM1* isoform 2) (12), a cell surface proteoglycan, as an IL-1RI co-receptor 13, 14. We have demonstrated that TILRR association with IL-1RI causes enhanced expression of IL-1RI and increased recruitment of the MyD88 adapter to the Toll/interleukin-1 receptor homology domain of IL-1RI (13). Further, we have shown that the resulting increase in signal amplification at the level of the receptor complex directs TILRR control of aberrant activation of NF-κB and inflammatory genes.

The present study investigates the role of TILRR in host defense and disease and demonstrates that TILRR is highly expressed in areas of vascular inflammation and lung fibrosis. Characterization of the inflammatory phenotype of our TILRR knockout (KO) mouse shows that changes in IL-1 receptor levels, signal transduction, and inflammatory gene activity, caused by genetic deletion, are consistent with molecular mechanisms of TILRR function identified in our published in vitro studies 13, 14. Using well-established models of vascular disease we demonstrate that TILRR KO and antibody blocking lead to reductions in monocyte activation, inflammatory gene activity, and disease progression, without causing development of vulnerable plaques. Taken together our results suggest that TILRR is a central regulator of inflammatory responses related to development of vascular disease, and that it may constitute a highly specific therapeutic target.

## Methods

### Mouse strains

#### TILRR^–/–^ mice

Mice were derived by the Center for Mouse Genome Modification (University of Connecticut, Farmington, Connecticut). The mouse TILRR transcript is encoded within a genomic region spanning across exons 24 to 36 of the *Frem1* gene, and the amino terminus of the TILRR protein from aa1-17 is encoded in the intron preceding exon 24 of *Frem1*. To create a null allele for TILRR, LoxP sites were inserted flanking exons 24 and 25 (Supplemental Figure 1). The targeting vector was prepared by recombineering according to Lee et al. (15). Briefly, we first retrieved approximately 12.8 kb of *Frem1* genomic sequence spanning 4 kb upstream of exon 24 to 3 kb downstream of exon 26 from the BAC, RP23-365E9, into PL253 containing the herpes simplex virus thymidine kinase negative selectable marker by recombineering (16). We inserted the 5′ LoxP site approximately 800 bp upstream of exon 24 followed by insertion of Frt-PGKneo-Frt-LoxP approximately 100 bp 3′ downstream of exon 25. The final vector contains 5′ and 3′ arms of 3 and 4 kb, respectively. The vector was linearized by digestion using NotI (restriction endonuclease, which recognizes the sequence 5’ GC/GGCCGC 3’) and purified, and subsequently electroporated into mouse embryonic stem cells derived from F1 (129Sv/C57BL/6J) blastocyst. Electroporated cells were cultured in the presence of G418 and gancyclovir (Life Technologies, Paisley, United Kingdom) 48 h post-electroporation. Drug-resistant colonies were picked and screened by long-range polymerase chain reaction (PCR) using primers corresponding to sequences outside the arms and specific to the 5′ and 3′ LoxP sites to identify targeted embryonic stem clones. These targeted embryonic stem clones were expanded and analyzed by long-range PCR for confirmation before using them for embryonic stem cell-morula aggregations (KSOM embryo culture medium, overnight, 37°C) and development of blastocysts for generation of chimeric animals.

Chimeric animals were bred with ROSA26-Flpe mice (Jax #003946, Jackson Laboratory, Bar Harbor, Maine) to remove the PGKneo cassette to generate the conditional knock-in mice, or Hprt-Cre mice (Jax #004302) to generate the global KO mice. C57BL/6J wild-type littermates were used as control mice.

#### Low-density lipoprotein receptor^–/–^/TILRR^–/–^ double KO mouse

Double knockout mice (TILRR^–/–^/LDLRR^–/–^) were bred using LDLR^–/–^(Jax 002207) and a conventional cross breeding strategy. Observed genotype ratios did not differ from those expected.

Apolipoprotein E (ApoE)–deficient (ApoE^–/–^) mice (Jax #2052) were obtained from the Jackson Laboratory.

#### PCR genotyping

Ear clippings were lysed in 50-μl alkaline lysis reagent (95°C, 2 h) before addition of neutralization reagent (50 μl), and 1 μl used for each PCR reaction. Each reaction used 12.5 μl BioMixRed (2×, Bioline), 0.6 μl of each primer (10 μM), and 1 μl of DNA in a total volume of 25 μl (Tables 1, 2, 3, and 4). Cycling conditions for TILRR KO reaction included an initial denaturation step (94°C, 3 min), followed by 33 cycles of denaturation (94°C, 30 s), annealing (55°C, 30 s) and extension (72°C, 15 s) with a final single extension step (72°C, 5 min). Cycling conditions for LDLR KO reaction were modified to include 40 cycles with an annealing temperature of 65°C.

**Table 1:** TILRR KO Genotyping Primers

Primer Name	Primer 5′ – 3′
Lox gt forward	CTG GGT GGC ATC TAG TAT TC
Lox gt reverse	CCC GAG ATT GCA GAG ATT CT
Frt gt forward	GAC GTC TGG AGA ACA CAA CA
Frt gt reverse	GAG TTC CTC TTT AGC TCT GC

KO = knockout; TILRR = Toll-like and interleukin-1 receptor regulator.

**Table 2:** Primer Combinations Used to Produce Expected Band Sizes for TILRR KO Genotyping

Primer Combination	Type	Expected Band Sizes
Lox forward and reverse	WT	233 bps
Lox forward and reverse	Floxed	325 bps
Frt forward and reverse	WT	25 bps
Frt forward and reverse	Floxed	223 bps
Lox forward and Frt reverse	WT	No product
Lox forward and Frt reverse	KO	273 bps
Lox forward, Frt forward, Frt reverse	WT	125 bps
Lox forward, Frt forward, Frt reverse	KO	273 bps

bps = base pairs; WT = wild-type; other abbreviations as in Table 1.

**Table 3:** LDLR KO Genotyping Primers

Primer Name	Primer 5′ – 3′
LDLR Common	CCA TAT GCA TCC CCA GTC TT
LDLR WT	GCG ATG GAT ACA CTC ACT GC
LDLR KO	AAT CCA TCT TGT TCA ATG GCC GAT C

KO = knockout; LDLR = low-density lipoprotein receptor; WT = wild-type.

**Table 4:** Primer Combinations Used to Produce Expected Band Sizes for LDLR KO Genotyping

Primer Combination	Type	Expected Band Size
LDLR Common and LDLR WT	WT	167 bps
LDLR Common and LDLR KO	KO	350 bps

To analyze TILRR/low-density lipoprotein receptor (LDLR) double KO mice, genotyping for LDLR and for TILRR were run in parallel. Expected band and sizes for TILRR/LDLR double KO mice were 273 bps using TILRR primers and 350 bps using LDLR primers.

Abbreviations as in Tables 1 and 2.

### In vivo disease models

#### Inflammatory responses

To assess the impact of TILRR KO on inflammatory responses, lipopolysaccharide (LPS) (10 mg/kg) (Enzo Life Sciences, Exeter, United Kingdom) was injected into the peritoneum of TILRR^–/–^ mice and control littermates to induce an acute inflammatory response and vehicle, phosphate-buffered saline (PBS), injections were used as control. Mice were sacrificed at 3 h post-injection and tissues prepared and inflammatory responses determined using in vivo models for atherosclerosis and fibrosis, as described subsequently.

#### Response to injury

This was assessed using carotid ligation, as previously (17). The right carotid artery of wild-type and TILRR KO mice was exposed and permanently ligated with a 6-0 suture just below the bifurcation. The contralateral artery received a sham ligation. Arteries were harvested from animals after 28 days following perfusion fixation. Arteries were embedded in paraffin wax, sectioned and stained for morphometric analysis and immunohistochemistry.

#### Atherosclerotic development

LDLR^–/–^, LDLR^–/–^/TILRR^–/–^, and ApoE^–/–^ mice were kept on a high-fat diet (18% lard fat, 1% cholesterol, and 0.5% sodium cholate) (Purina Mills, St. Louis, Missouri) for 12 weeks, from 8 weeks of age, and were housed with a 12-h light–dark cycle at 22°C.

To assess the impact of TILRR antibody blocking on development of atherosclerosis, ApoE^–/–^ mice were injected with vehicle alone (PBS) or nonspecific immunoglobulin (IgG) (control mice), or with an antipeptide antibody targeting the TILRR functional site, described subsequently (intravenous 2×/week, 130 mg/kg) (custom made, Eurogentec Ltd., Southampton, United Kingdom). Antibody injections were started at 8 weeks of age and continued throughout the 12-week experiment. Antibody concentration was determined based on initial pilot experiments.

Atherosclerosis extent and composition was determined as described previously and outlined subsequently (18).

#### Lung fibrosis

ApoE^–/–^ mice were kept on a high-fat diet and injected with anti-TILRR antibody, or with PBS or nonspecific IgG (control mice), as described previously. Lung tissue was prepared, stained, and analyzed as outlined subsequently.

Mice were sacrificed by exsanguination under pentobarbital anesthesia. All experiments were performed in accordance with UK Home Office legislation under the 1986 Animals (Scientific Procedures) Act, and covered by licenses PPL 70/7992 and PPL 30/3531.

### Peptide antibody development

Earlier studies using alanine-scanning mutagenesis of conserved residues within the TILRR core protein identified a functional site, which caused a 60% reduction in inflammatory responses without affecting IL-1-induced antiapoptotic signals 13, 14. A peptide (FDSTDLSQRKLRTRG) containing the functional site at residue D448 was used to develop a blocking peptide antibody (anti-rabbit, custom, Eurogentec). The antibody was tested in in vitro assays to determine effects on IL-1-induced activation of NF-κB regulated inflammatory responses and antiapoptotic signals (Supplemental Figure 2). Cultures were pre-incubated with the peptide antibody (0, 10, 100, 200 ng/ml) for 2 h before stimulation with IL-1 (10^−9^ M, 6 h), and effects on inflammatory responses analyzed by reporter assay using IL-8 as readout, and antiapoptotic signals determined by monitoring activation of caspase-3 and -7. Control cultures were incubated with a nonspecific IgG.

### Tissue and sample preparation

#### Protein, DNA, and RNA extraction from spleen tissue

Spleen tissue taken from TILRR^–/–^ and wild-type, LPS-injected mice were flash frozen with liquid nitrogen and pulverized using pestle and mortar. The disrupted tissue was lysed in Buffer RLT (AllPrep Kit, Qiagen, Manchester, United Kingdom) containing 1% β-mercaptoethanol and homogenized by passing through a 25-gauge needle 10 times. Samples were then processed using the AllPrep Kit according to the manufacturer’s instructions. Proteins were dissolved in 5% (w/v) sodium dodecyl sulfate (SDS) containing 1× protease inhibitor cocktail (Roche Diagnostics, Burgess Hill, United Kingdom) by manual mixing and heating to 37°C. Total protein was quantified by bicinchoninic acid assay (Pierce Chemical, Dallas, Texas). Total DNA and RNA was determined using the NanoDrop 1000 machine (Thermo Fisher Scientific, Loughborough, United Kingdom).

#### Isolation of bone marrow cells

Femurs of TILRR^–/–^ and wild-type mice were dissected and adherent tissue removed. Bones were then washed in 70% ethanol solution and Dulbecco’s modified Eagle’s medium containing 10% fetal calf serum and 1% penicillin streptomycin (Thermo Fisher Scientific). The epiphysis was removed and the bone flushed with a 21- or 26-gauge hypodermic needle and syringe filled with Dulbecco’s modified Eagle’s medium to extrude the bone marrow into a Petri dish. The bone marrow was homogenized by gentle pipetting and the cells counted using a hemocytometer. Cells were centrifuged (750 to 1,000 *g*, 5 min) and resuspended in bone marrow–derived macrophages differentiation medium (10% fetal calf serum, 20 ng/ml M-CSF) for 7 days. Cells were seeded at 5 × 10^6^ in 10 cm dish with bone marrow–derived macrophages differentiation medium overnight or frozen in liquid nitrogen.

### Morphology

#### Tissue preparation

##### Vascular and lung tissue

After perfusion with ice-cold PBS, the aortic root, brachiocephalic and carotid arteries, and lungs were fixed in 10% buffered formalin (5 h, 4°C), embedded in paraffin, and 5-μm sections mounted on polylysine-coated glass slides (Gerhard Menzel GmbH, Braunschweig, Germany).

#### Histology

Serial sections of the aortic root and the brachiocephalic artery, 90 μm apart, were dewaxed in xylene and rehydrated in a graded series of ethanol (100%, 90%, 70%, 50%), washed in water and stained with Alcian blue and elastic van Gieson as described (19). Briefly, sections were dewaxed in xylene and rehydrated through graded alcohols and oxidated using potassium permanganate (0.25%, 30 min), bleached in oxalic acid (1%) and nuclei counterstained with Carazzi’s hematoxylin (2 min), followed by rinses in acid alcohol (1% hydrochloric acid, 70% ethanol, 5 min). Following staining with Alcian blue (1%) in aqueous acetic acid (3%, pH 2.5, 5 min) sections were washed in water and ethanol (95%), stained with Miller’s elastin (30 min), rinsed in ethanol (95%) and distilled water, and incubated with Curtis’s modified van Gieson stain (6 min). Sections stained with Trichrome were incubated in Bouin’s solution (10 min, 58°C) and Weigert’s iron hematoxylin solution (5 min), washed (30 s), stained with Trichrome solution (15 min) (Sigma-Aldrich, St. Louis, Missouri), and placed in acetic acid (10 s, 0.5%). For hematoxylin and eosin staining, dewaxed sections were immersed in hematoxylin (4 min) and rinsed in tap water, immersed in eosin (1 to 2 min), and mounted using ProLong Gold Antifade (Life Technologies).

For collagen staining slides were incubated with picrosirius red (0.1%, 1 h) (Sigma-Aldrich) or Martius scarlet blue as described (20) and levels of plaque collagen expressed as a percentage of the total surface area. Briefly, sections were dewaxed and rehydrated in xylene and graded alcohols and stained with 1% (w/v) Celestine blue (5 min) and counterstained with Harris’s hematoxylin (5 min). After rinsing in tap water and acid alcohol, sections were rinsed in hot tap water and ethanol (95%), and stained with 0.5% (w/v) Martius yellow and 2% (w/v) phosphotungstic acid in 90% (v/v) ethanol (2 min), and immersed in ponceau de xylidine solution (1% [w/v] ponceau de xylene/2% [v/v] glacial acetic acid, 10 min). The stain was differentiated using phosphotungstic acid (1%, 5 min) and sections stained with methyl blue (5% [w/v] methyl blue in 10% [v/v] glacial acetic acid, 10 min), washed in acetic acid (1%, 10 min), and rehydrated through graded series of alcohols and xylene (2) and mounted using DPX (VWR International, Lutterworth, United Kingdom).

En face staining of the whole aorta was carried out as described (21). In brief, the aortas were perfused with PBS and 10% (v/v) formalin, dissected, fixed in 10% (v/v) formalin, and stored in PBS (overnight, 4°C). The aorta was opened longitudinally and stained with Oil Red O stain (Sigma Aldrich), rinsed in isopropanol (60% [v/v]), stained with 0.3% (w/v) Oil Red O stain (Sigma-Aldrich) in PBS (30 min), de-stained in isopropanol (60% [v/v] 20 min) and washed in distilled water, and images of the exposed luminal portion of the aorta taken using light microscopy.

### Immunohistochemistry

Sections of vascular samples were prepared as previously (22). Briefly, formalin-fixed, paraffin-embedded samples were deparaffinized and rehydrated through decreasing concentrations of ethanol. Sections were treated with hydrogen peroxidase (3% in PBS) and sodium citrate solution (10 mM, pH 6.0, Alfa Aesar, Ward Hill, Massachusetts) (20 min, 95°C) to block endogenous peroxide and induce antigen retrieval, washed in PBS, and blocked with 10% goat serum or 1% (w/v) nonfat milk in PBS (30 min). Sections were incubated with primary antibodies (PBS, 1% bovine serum albumin, 4°C, overnight); affinity purified nonblocking rabbit peptide polyclonal anti-TILRR (1:50, peptide GNERYFWIGL, custom made, Eurogentec), mouse monoclonal smooth muscle cell anti-α-actin (1:100, Dako, Glostrup, Denmark), or rat monoclonal anti-Mac-3 (anti-mouse CD107b; 1:100 or 1/150, Pharmingen, San Diego, California), followed by incubation with appropriate secondary antibody, as required: goat anti-rabbit DyLight 550- or 633-conjugated antibody, goat anti-mouse DyLight 488–conjugated antibody, or goat anti-rat DyLight 488–conjugated antibody (all at 1:200, ImmunoReagents, Raleigh, North Carolina), or with biotinylated antibodies, followed by a 30-min incubation with an avidin-biotin complex (Vectastatin Elite ABC kit, Vector Laboratories, Peterborough, United Kingdom) and 3,3’-diaminobenzidine (DAB), using well established procedures. Nuclei were counterstained with Carazzi’s hematoxylin or 4’,6-diamidino-2-phenylindole (DAPI).

### Morphometry

Images of sections stained for collagen, vascular smooth muscle cell α-actin and Mac-3 were analyzed with NIS-Elements software (Nikon UK Limited, Kingston Upon Thames, United Kingdom). The size of the plaque area was expressed as micrometers squared. The amount of collagen, vascular smooth muscle cells and macrophages in plaques, and vascular smooth muscle cells in the medial area were quantified using a color threshold. For quantification of pulmonary fibrosis, the number of granulomas and inducible bronchus-associated lymphoid tissue (iBALT), and the level of pulmonary vascular remodeling, 3 Trichrome-stained sections 200 μm apart were analyzed for each lung. Assessment of pulmonary fibrosis was made, as previously, using 10 images per lung section, excluding areas with tracheal or bronchial tissue, and fields graded using the Ralf-Harto Hübner scale 23, 24. Pulmonary muscularization was assessed by measuring the medial area of vessels with a diameter above 100 μm.

### Microscopy

Microscopy was carried out using a Nikon E600 microscope (Nikon UK Limited) and quantitation made using NIS-Elements software.

### Flow cytometry

Blood (100 μl) from wild-type and TILRR KO mice was collected 3 h after injection with LPS (10 mg/kg) or PBS (control) into 1 ml of PBS containing 5 μM ethylenediaminetetraacetic acid (EDTA) and erythrocytes were lysed using red blood cell lysis buffer. Fc receptors were blocked by pre-incubating with 0.5 to 1 μg of purified anti-mouse CD16/CD32 antibodies (eBioscience, San Diego, California) for 10 min at 4°C before staining. Cells were stained (30 min, 4°C) with a combination of fluorochrome-conjugated antibodies: Pacific Blue anti-mouse CD11b, PE/Cy7 anti-mouse Ly-6G/Ly-6C, and PE anti-mouse F4/80 (1 mg/100,000 cells) (Biolegend, San Diego, California). For live/dead discrimination LIVE/DEAD Fixable Blue Dead cell stain kit, for ultraviolet excitation (Life Technologies) was used. After fixation O/N in paraformaldehyde (1%, 4°C) cells were analyzed on BD LSRII Flow Cytometer. For color compensation AcCTM anti-rat/hamster bead kit or AcCTM anti-mouse bead kit in combination with corresponding antibodies and ArC Amine Reactive Compensation Bead kit with LIVE/DEAD Fixable Blue Dead cell stain kit were used.

### Cell culture

#### Mouse cell lines

RAW 264.7 cells (murine monocyte/macrophage cell line, ATCC) were propagated in Dulbecco’s modified Eagle’s medium containing 10% fetal calf serum, and were used in initial functional in vitro assays.

#### Mouse primary cells

Bone marrow cells from TILRR^–/–^ and wild-type mice were cultured using standard conditions of 5% CO_2_ at 37°C in 10% fetal calf serum Dulbecco’s modified Eagle’s medium supplemented with 20 ng/ml macrophage colony-stimulating factor for 7 days to stimulate differentiation into bone marrow–derived macrophages. A total of 2 × 10^5^ cells were seeded in 6-well plates and stimulated the following day with 1 nM recombinant IL-1β (R&D systems, Abingdon, United Kingdom) for required time. Cells were lysed in SDS lysis buffer (1% v/v Triton X, 0.5% [w/v] SDS and 0.1% dimethoxy-4-chloroamphetamine [w/v]). Lysates were vortexed and incubated on ice for 20 min, followed by 20 min centrifugation at 13,000 rpm (4°C).

### Macrophage differentiation

RAW 264.7 cells were differentiated to M1 or M2 macrophage subpopulations using conventional procedures by incubating with LPS (100 ng/ml) and interferon-gamma (50 ng/ml, R&D systems) or with IL-4 (10 ng/ml), respectively, for 24 h. Total RNA was isolated using the RNeasy extraction kit (Qiagen) and complementary DNA generated by reverse-transcription PCR using a High Capacity RNA-to-cDNA Kit (Applied Biosystems, Warrington, United Kingdom) and analyzed by quantitative PCR (qPCR).

### Western blotting

Extracted samples were separated by SDS–polyacrylamide gel electrophoresis (4% to 12%) and transferred to nitrocellulose membranes (GE Healthcare, Cardiff, United Kingdom). For analysis of signaling intermediates, membranes were blocked using Odyssey blocking buffer (30% in PBS, Li-Cor, Cambridge, United Kingdom) or milk Tris-buffered saline (5% milk, Tris-buffered saline, 0.1% Tween). TILRR was detected by an affinity-purified specific nonblocking rabbit peptide anti-TILRR antibody, as previously (1:1,000, custom made, Eurogentec) 13, 14. Incubation with antibodies against IL-1RI (1:500, Abcam, Cambridge, United Kingdom), tumor necrosis factor receptor 1 (TNFRI) (1:250, Abcam), TLR2 (1:1,000, Novus Biologicals, Abingdon, United Kingdom), TLR4 (1:1,000, Novus Biologicals), inhibitor kappa B alpha (IκBα) (1:1,000, Santa Cruz Biotechnology, Heidelberg, Germany), phospho-IκBα (1:1,000, Cell Signaling Technology, Leiden, the Netherlands), and β-actin (1:1,000, Santa Cruz) was followed by probing with a relevant secondary antibody and developed using LI-COR Odyssey (Li-COR, Biosciences, Lincoln, Nebraska) and quantitation using ImageJ (64-bit Java) (National Institutes of Health, Bethesda, Maryland).

### Luciferase assay

Cells were seeded in 96-well plates (Nunc), and transfected with herpes simplex virus thymidine kinase promoter with Renilla luciferase expression plasmid (75 ng TKRL) and IL-8-luciferase (100 ng) using Polyfect (Qiagen) according to the manufacturer’s instructions. Twenty-four hours post-transfection, cells were pre-incubated the anti-TILRR peptide antibody, as described previously, and stimulated with IL-1β (10^−10^ M or 10^−9^ M, 6 h). Luciferase assay was carried out using the Dual-Luciferase Reporter Assay System (Promega, Southampton, United Kingdom), according to the manufacturer’s instructions. Briefly, cells were lysed in 50 μl passive lysis buffer during shaking (15 min). Lysate (15 μl) and firefly luciferase (25 μl) were added to each well and luminescence measured using a Varioskan plate reader (Thermo Scientific), and levels of the TK-RL control plasmid determined after addition of Stop & Glo (25 μl).

### Caspase assay

Caspase activity was determined using the caspase-3 and -7 Glo Luminometric Assay (Promega) according to the manufacturer’s protocol. Seventy-two hours after seeding, cells were washed with PBS and treated with tumor necrosis factor alpha (10 ng/ml) plus cycloheximide (10 μg/ml) for 3 h to induce apoptosis ± prior IL-1 (1 nM, 3 h) stimulation. Cultures were incubated in the presence of caspase-3 and -7 Glo reagent (50 μl/well, 2 h), reactions transferred to a white walled plate and the luminescence measured on a Varioskan plate reader.

### Enzyme-linked immunosorbent assay

Cells were incubated with the TILRR peptide antibody or with an IgG control, and stimulated with IL-1, as previous. IL-6 protein levels were measured using a human IL-6 DuoSet enzyme-linked immunosorbent assay, according to the manufacturer’s instructions.

### TILRR small interefering RNA

Twenty-four hours after plating, HeLa cells were transfected with either ON-TARGETplus Nontargeting small interfering RNA (50 nM, Fisher) or custom TILRR small interfering RNA (50 nM, Eurogentec), using Dharmafect1 (24 μl, Dharmacon, Lafayette, Colorado), demonstrated to reduce TILRR expression by on average 70% (13).

### Microarray analysis

Total RNA was harvested from spleen and blood monocytes of TILRR^–/–^ and wild-type C57BL/6J mice using the RNeasy kit (Qiagen) and RNA quality and concentration determined using the 2100 Bioanalyzer (Agilent Technologies, Cheadle, United Kingdom). Samples were labeled and hybridized using the Affymetrix Gene Chip Hybridization platform (Affymetrix, High Wycombe, United Kingdom) and scanned using the Illumina HiScan SQ in the Sheffield Microarray/Genomics Core Facility. Gene expression profiles were analyzed using R Bioconductor 2.14 (Bioconductor) and DAVID 6.7 (National Institute of Allergy and Infectious Diseases [NIAID], NIH) 25, 26, and data expressed as log2-fold change of activity in TILRR^–/–^ relative to wild-type.

### qPCR analysis

RNA was prepared from extracted spleen tissue from TILRR^–/–^ and wild-type mice, injected with LPS (10 mg/kg, Enzo Life Sciences), as described previously. Extracted and cultured bone marrow–derived macrophages were lysed using Buffer RLT and total RNA isolated using RNeasy kit (Qiagen). One microgram of total RNA was reverse transcribed to complementary DNA (cDNA) (Applied Biosystems) and 1 μl was added to standard Taqman qPCR reaction (Applied Biosystems) containing relevant TaqMan MGB probes (murine probes): glyceraldehyde 3-phosphate dehydrogenase (GAPDH), chemokine ligand 2 (CCL2), C-X3-C motif chemokine ligand 1 (CX3CL1), tumor necrosis factor alpha (TNFα), and interleukin (IL)-6 (Thermo Fisher Scientific).

Venous blood samples taken sequentially from patients on the day of presentation (day 1) and days 7 and 90 following ST-segment elevation myocardial infarction (STEMI) or non–ST-segment elevation myocardial infarction (NSTEMI), and venous blood samples from healthy volunteers were screened for TILRR expression, using an N-terminal probe (Thermo Fisher Scientific). RNA was purified from human blood stored in Tempus tubes using Maxwell 16 miRNA Blood Kit (Promega) and the Maxwell 16 Instrument (Promega) as per manufacturer’s instructions. Results were analyzed by RQ Manager analysis software (Thermo Fisher Scientific). Critical threshold values were normalized to endogenous GAPDH control. Readings using a C-terminal probe for *FREM1* (Thermo Fisher Scientific) were subtracted from the data.

### Agent-based modeling

Simulations comparing activation in the presence and absence of TILRR amplification were carried out using the agent-based modeling representing activation of the NF-κB network, as described 27, 28, 29, 30, 31, 32.

### Protein structure modeling and docking

The tertiary structure model of TILRR (NP_001171175.1) was built using multiple-threading alignments and iterative fragment assembly in the de novo I-Tasser Zhang Server (33). The extracellular domain of IL-1RI was generated in Swiss-Model (34), using IL-1RI from the resolved crystal structure complex (PDB:4DEP) (35). The protein-docking model was predicted using generated PDB files in Gramm-X (36). Protein tertiary structure models were viewed and modified in MolSoft ICM Browser and protein structure template quality scored 37, 38.

### Statistical analysis

Data showing normal distribution were analyzed by Student's *t* test or 1-way analysis of variance (Prism 6, GraphPad Software, San Diego, California), and using Dunnett’s test or Tukey’s post hoc test for multiple comparisons, and are presented as mean ± SEM.

All in vitro and ex vivo experiments included triplicate samples and statistical analysis was based on 3 to 5 independent experiments. Morphological data were obtained from 5 to 12 control animals and 5 to 15 experimental animals. Statistical analysis was based on average readings for each animal, which were calculated based on 3 to 7 independent measurements from serial sections, 90 or 200 μm apart.

Specific information relating to each figure and p values are included in the respective legends.

## Results

### The TILRR KO mouse exhibits impaired control of NF-κB and reduced gene activity

**Figure 1:**
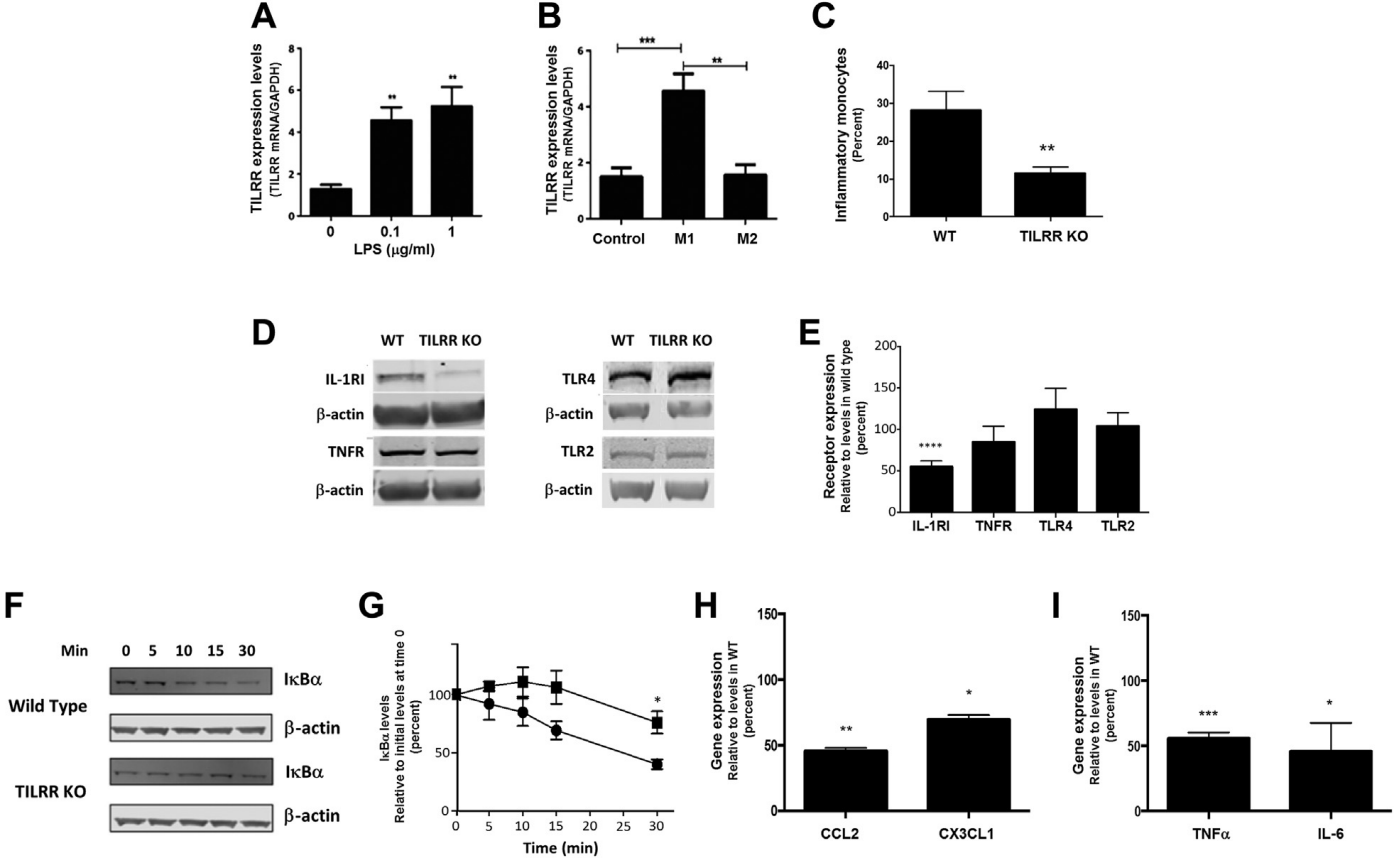
TILRR Controls IL-1RI Levels, Signal Amplification, and Gene Activity **(A)** Quantitative polymerase chain reaction (qPCR) of TILRR expression in raw cells, stimulated with lipopolysaccharide (LPS) (6 h) over a range of concentrations, as indicated. Data are expressed relative to levels of glyceraldehyde 3-phosphate dehydrogenase (GAPDH) and show mean ± SEM. n = 3.0 μg/ml LPS versus 0.1μg/ml LPS; 0 μg/ml LPS versus 1 μg/ml LPS, **p < 0.01. **(B)** qPCR of Toll-like and interleukin-1 receptor regulator (TILRR) expression in M1- and M2-like macrophages and undifferentiated cultures (control). Data are expressed relative to levels of GAPDH and show mean ± SEM. n = 3, **p < 0.01,***p < 0.001. **(C)** Fluorescence-activated cell sorting analysis of inflammatory monocytes from wild-type (WT) and TILRR knockout (KO) mice injected with LPS (10 mg/kg). Data are expressed as percent of total monocyte levels and show mean ± SEM. n = 6 WT, n = 9 TILRR KO. Levels in TILRR KO mice versus levels in WT mice, **p < 0.0088. **(D)** Western blots of interleukin-1 receptor type I (IL-1RI), tumor necrosis factor receptor (TNFR), Toll-like receptor 4 (TLR4), and Toll-like receptor 2 (TLR2) expressions in spleen from WT and TILRR KO mice. **(E)** Quantitation of Western blots as in **D** showing receptor levels in TILRR KO cells relative to levels in WT cells, using to β-actin as loading control. The graph shows mean ± SEM, n = cells from 4 to 6 WT mice and 4 to 6 TILRR KO mice for each receptor. Levels of IL-1RI, TNFR, TLR4, and TLR2 in TILRR KO mice are expressed as percent of levels of the respective receptor in WT mice, IL-1RI expression in TILRR KO mice versus IL-1RI expression in WT mice. ****p < 0.0001. **(F)** Western blot analysis of interleukin (IL)-1–induced (10^−9^ M) inhibitor kappa B alpha (IκBα) degradation in bone marrow–derived macrophages from WT and TILRR KO mice. **(G)** Quantitation of Western blots as in **F** showing IL-1–induced IκBα degradation in WT **(circles)** and TILRR KO **(squares)** cells. Data are expressed as percent of IκBα levels in unstimulated WT and TILRR knockout cells, respectively (time 0) and show mean ± SEM. n = peripheral blood mononuclear cells from 4 WT mice and 4 TILRR KO mice. Levels in TILRR KO cells versus levels in WT cells at 30 min. *p = 0.0124. **(H)** qPCR of bone marrow–derived macrophages from TILRR KO mice shows reductions in chemokine ligand 2 (CCL2) and C-X3-C motif chemokine ligand 1 (CX3CL1). Data are expressed as percent activation of the respective gene in cells from WT mice and show mean ± SEM. n = cells from 4 TILRR KO mice and 4 WT mice. CCL2 expression in TILRR KO cells versus expression in WT cells, **p = 0.0022; CX3CL1 expression in TILRR KO cells versus expression in WT cells, *p = 0.0482. **(I)** qPCR of spleen samples from TILRR KO mice shows reductions in proinflammatory cytokines tumor necrosis factor (TNF)-α and IL-6. Data are expressed as percent activation of the respective gene in spleen samples from WT mice, and show mean ± SEM. n = 5 WT and 5 TILRR KO mice. TNF-α expression in TILRR KO cells versus expression in WT cells, ***p = 0.0009. IL-6 expression in TILRR KO cells versus expression in WT cells, *p = 0.0236.

Initial in vitro experiments using qPCR demonstrated that LPS stimulation induced a concentration-dependent increase in TILRR expression in mouse macrophages, and showed that expression levels in M1 macrophages were increased by about 3-fold, from 1.50 ± 0.63 relative to GAPDH in control cells to 4.55 ± 0.82, whereas levels in the M2 phenotype were similar to levels in undifferentiated cultures (1.57 ± 0.44) (Figures 1A and 1B). The relevance of TILRR expression in monocyte activation was supported by subsequent in vivo experiments using a TILRR KO mouse (Supplemental Figure 1), which demonstrated that TILRR deletion caused a reduction in activated blood monocytes from 28.13 ± 5.04% to 11.34 ± 1.81% (Figure 1C).

Characterization of the inflammatory phenotype of the TILRR KO mouse demonstrated effects on IL-1 receptor levels, signal transduction, and gene regulation. Western blot analysis of spleen samples showed that IL-1RI expression in TILRR KO mice was significantly reduced, with levels corresponding to 55 ± 7% of expression wild-type mice, whereas levels of tumor necrosis factor receptor (85 ± 17%), Toll-like receptor 4 (124 ± 23%), and Toll-like receptor 2 (104 ± 14%) were not significantly different from control mice (Figures 1D and 1E). Subsequent experiments assessed effects of genetic deletion of TILRR on IL-1–induced activation of NF-κB, using degradation of the inhibitor IκBα as readout. Thirty minutes of IL-1 stimulation of peripheral blood mononuclear cells (PBMCs) from wild-type mice caused, as expected, a successive reduction in IκBα to levels corresponding to 40 ± 4% of levels present before activation (Figures 1F and 1G). In comparison, inhibitor levels in cells from TILRR KO mice, subjected to the same treatment, were significantly higher, corresponding to 76 ± 9% of initial levels. The reduction in inhibitor degradation from 60% in control PBMCs to 24% in the TILRR KO cells is consistent with impaired induction of NF-κB, and agrees with results from our earlier in vitro experiments, demonstrating that inhibiting TILRR function significantly reduces IL1-induced activation of the pathway 13, 14, 27. In silico analysis using an agent-based model of NF-κB regulation in TILRR KO cells predicted the observed changes in receptor levels and signal amplification to have significant effects on activation of inflammatory genes (Supplemental Figures 3A and 3B). Microarray analysis of blood and spleen samples from LPS-injected TILRR KO mice demonstrated pronounced reductions in activation of proinflammatory regulators compared with levels in wild-type mice (Supplemental Figure 4). Results were confirmed by qPCR analysis. Figure 1H shows reduced induction of chemokine ligand 2 (CCL2) (45.0 ± 2.4%) and C-X3-C motif chemokine ligand 1 (CX3CL1) (69.55 ± 3.46%) in bone marrow–derived macrophages from TILRR KO mice compared with levels measured in wild-type cells. Similarly, analysis of spleen samples from TILRR KO mice demonstrated pronounced reductions in cytokines, including TNFα (55.8 ± 4.5%) and IL-6 (45.8 ± 22.0%) (Figure 1I).

Subsequent studies assessed the role of TILRR in cardiovascular disease and lung fibrosis, and evaluated its potential as a therapeutic target.

### Response to injury is significantly reduced in the TILRR KO mouse

**Figure 2:**
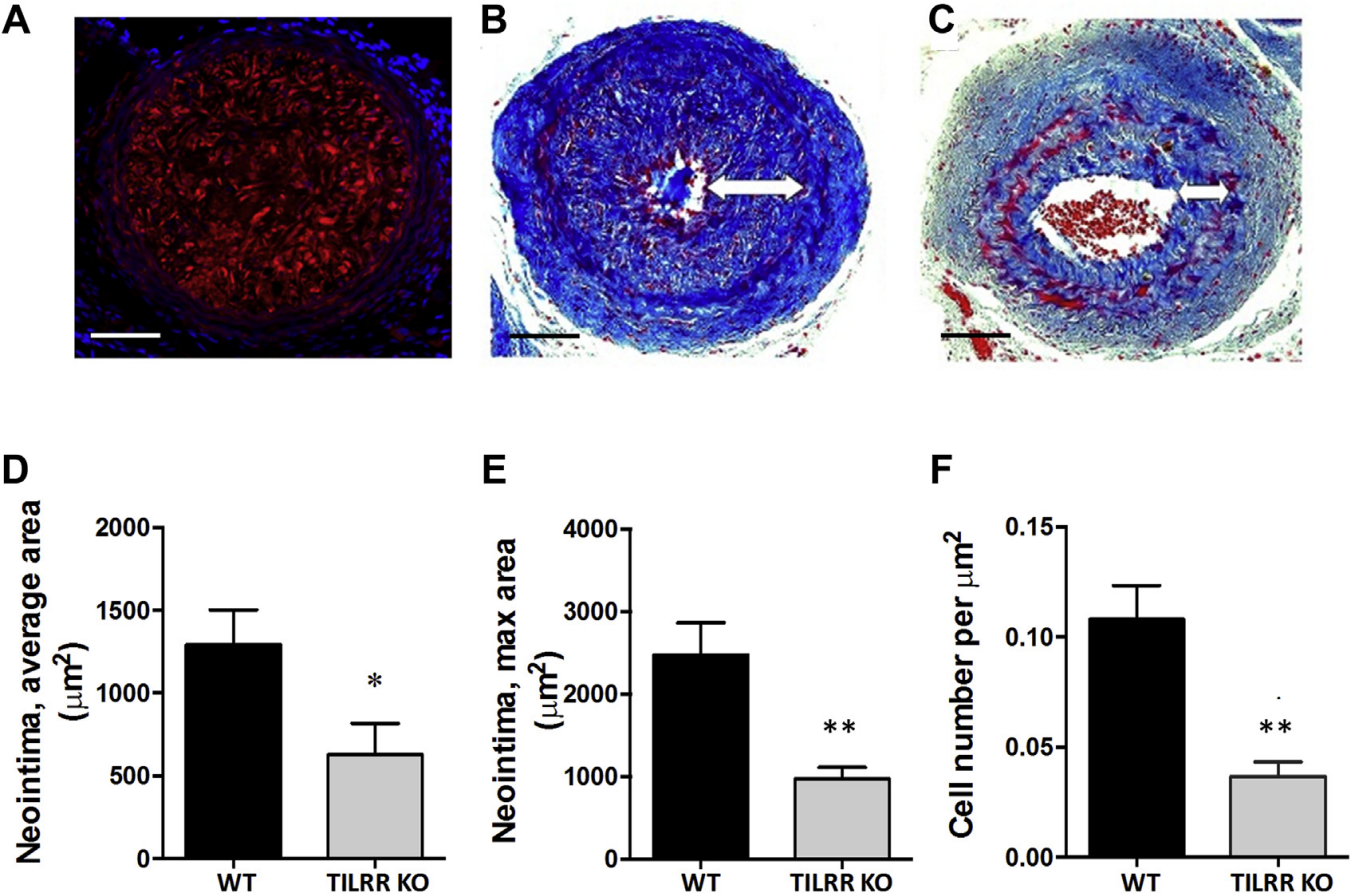
Response to Injury Is Significantly Reduced in the TILRR-Knockout Mouse **(A)** Immunohistochemistry of a cross-section of a carotid artery in a WT mouse subjected to 4 weeks of ligation. Staining using a nonblocking anti-TILRR antibody followed by a DyLight Fluor-conjugated secondary antibody shows high levels of TILRR expression **(red)**. Scale bar = 150 μm. Trichrome staining of cross-sections of carotid arteries after 4 weeks of ligation in **(B)** a WT mouse and **(C)** a TILRR KO mouse. **Arrows** indicate thickness of the neointima in response to treatment. Scale bar = 150 μm. **(D to F)** Quantitation of measurements from cross sections as in **B** and **C** shows **(D, E)** a significant reduction in the thickness of the neointima and **(F)** a decrease in cell number in the adventitia in TILRR KO mice compared with levels in WT mice. Data are expressed as **(D, E)** area (μm^2^) and **(F)** cell number per μm^2^ and show mean ± SEM. n = 6 WT mice, 7 TILRR KO mice. TILRR KO mice versus WT mice, **(D)** *p = 0.038, **(E)** **p = 0.0045, **(F)** **p = 0.0014. Abbreviations as in Figure 1.

Initial experiments used an in vivo carotid ligation model to monitor responses to tissue injury in the TILRR KO mouse. Immunostaining of arterial cross sections from wild-type mice demonstrated high levels of TILRR expression at the ligation site (Figure 2A). A pronounced thickening of the neointimal layer of the vessel in control animals, induced in response to ligation, was significantly reduced in TILRR KO mice subjected to the same treatment (Figures 2B and 2C). Quantitation revealed a reduction in the average Neointima of about 50% (from 1,292 ± 211 μm^2^ to 629 ± 188 μm^2^) and a decrease in the maximum thickness of about 60% (from 2,476 ± 388 μm^2^ to 971 ± 141 μm^2^) (Figures 2D and 2E). Further, the cell density in the adventitia was significantly reduced, from 0.108 ± 0.015 cells/μm^2^ in the wild-type mice to 0.04 ± 0.01 cells/μm^2^ in the TILRR KO mice (Figure 2F).

### TILRR is highly expressed in PBMCs from patients diagnosed with myocardial infarction and in the atherosclerotic plaque

**Figure 3:**
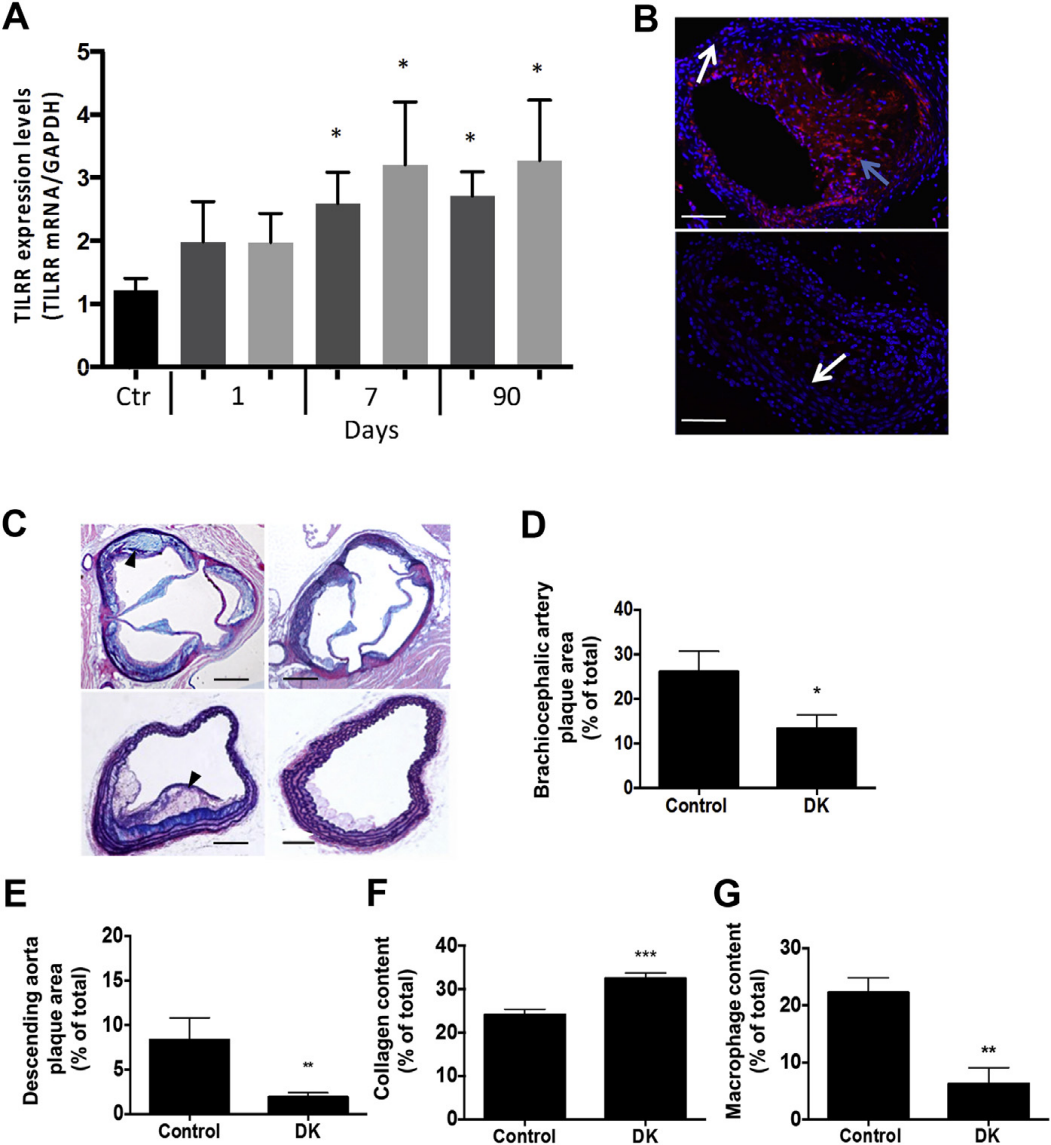
TILRR KO Significantly Reduces Progression of Atherosclerosis in LDLR^–/–^ TILRR^–/–^ (DK) Mice **(A)** TILRR expression levels in blood samples from patients with myocardial infarction (ST-segment elevation myocardial infarction [STEMI], **light gray bar**; non–ST-segment elevation myocardial infarction [NSTEMI], **dark gray bar**) at diagnosis, day 1, and at days 7 and 90. Values are mean ± SEM. n = 6 to 11 triplicate samples per group and time point. TILRR expression levels in each group of patient samples are compared with levels in healthy control samples **(black bar)**. STEMI day 7, *p = 0.0442; day 90, *p = 0.0329. NSTEMI day 7, *p = 0.0445; day 90, *p = 0.0405. **(B)** Immunohistochemistry of an atherosclerotic plaque in the brachiocephalic artery, using a nonblocking anti-TILRR antibody, followed by a DyLight Fluor-conjugated secondary antibody, shows increased levels of TILRR expression within the lesion **(top image, blue arrow)**, with low levels in the surrounding area **(top image, white arrow)** and in a healthy vessel (**bottom image**, **white arrow**). TILRR **(red)**, nuclear stain (4'6,-diamidino-2-phenylindole, **blue**). Scale bar =150 μm. **(C)** Cross-sections of the aortic root **(top)** and brachiocephalic arteries **(bottom)** of LDLR^–/–^ mice (control, **left**) and LDLR^–/–^/TILRR^–/–^ mice (double knockout [DK], **right**), stained with Alcian blue & van Gieson. Scale bar = 500 μm (aortic root), 200 μm (brachiocephalic artery). **Arrowheads** point at plaques in arteries from control mice. **(D, E)** Quantitation, using images such as in **C**, shows levels of atherosclerotic plaques in **(D)** the brachiocephalic artery and **(E)** the descending aorta in DK mice and control mice. Data are expressed as percent of total area and show mean ± SEM. **(D)** n = 12 control mice, 15 DK mice. **(E)** n = 6 control mice, 9 DK mice. Levels in DK mice versus levels in control mice, **(D)** *p = 0.0396; **(E)** **p = 0.004. **(F)** Quantitation of collagen content following staining of sections using Martius scarlet blue. Data are expressed as percent area stained for collagen in control and DK mice and show mean ± SEM. n = 6 control mice, 6 DK mice. Levels in DK mice versus levels in control mice, ***p = 0.0004. **(G)** Quantitation of macrophage levels in plaques from control and DK mice following staining of sections using anti-Mac-3. Data are expressed as percent stained area and show mean ± SEM. n = 5 control, 5 DK mice. Levels in DK mice versus levels in control mice, **p = 0.003. Abbreviations as in Figure 1.

Peripheral blood mononuclear cells from patients with STEMI and NSTEMI types of acute myocardial infarction showed a progressive increase in TILRR expression. TILRR messenger RNA levels in control samples, normalized to the internal control, GAPDH, as described in the Methods, were on average 1.22 ± 0.18 (Figure 3A). Levels in samples from patients diagnosed with STEMI-type acute myocardial infarction were 1.97 ± 0.41, 3.20 ± 1.00, and 3.27 ± 0.96 on days 1, 7, and 90 after diagnosis, respectively. Corresponding values for patients with NSTEMI-type myocardial infarction were 1.98 ± 0.64, 2.59 ± 0.50, and 2.71 ± 0.38.

Immunostaining of the aortic root and the brachiocephalic artery from ApoE^–/–^ and LDLR^–/–^ mice, fed a high-fat diet, demonstrated high levels of TILRR expression within vascular lesions (Figure 3B, top image, blue arrow). In contrast, levels were low or undetectable in tissue surrounding the plaque and in unaffected vessels (Figure 3B, white arrows, top and bottom images).

### TILRR KO reduces the size and changes the characteristics of atherosclerotic lesions

A subsequent set of experiments used LDLR^–/–^/TILRR^–/–^ DK mice to evaluate the impact of genetic deletion of TILRR on development and characteristics of atherosclerosis. Histological analysis revealed pronounced reductions in plaque development in the aortic root and the brachiocephalic artery (Figure 3C). Quantitation showed that plaque levels in the brachiocephalic artery, which corresponded to 26.29 ± 5.23% of the cross-sectional area of the vessel in the LDLR^–/–^ mice, used as control mice, was reduced to 15.92 ± 2.88% in the DK mice (Figure 3D). Further, plaque levels in the descending aorta were reduced from 8.3 ± 2.5% to 1.9 ± 0.5% of the cross-sectional area of the vessel (Figure 3E). Characterization of the plaques revealed a 35% increase in collagen content in lesions from the DK mice (32.50 ± 1.29%) compared with control mice (24.17 ± 1.22%) and a 75% reduction in macrophage levels from 22.25 ± 2.59% to 6.25 ± 0.84% (Figures 3F and 3G).

### Blocking TILRR function reduces progression of atherosclerosis

A subsequent set of experiments used the ApoE^*–/–*^ mouse model to establish the impact of antibody blocking of TILRR function on progression of atherosclerosis. These studies used a blocking peptide antibody, which was developed to target the TILRR functional site at residue D448, identified in our earlier study (13). Three-dimensional modeling of the receptor complex predicted the residue to be located in the area of TILRR–IL-1RI association (Supplemental Figures 2A and 2B). In vitro studies, carried out to assess the impact of the peptide antibody on IL-1–induced responses, demonstrated a concentration-dependent reduction in activation of inflammatory genes (Supplemental Figure 2C). Levels, expressed relative to activities in cultures incubated with a nonspecific IgG control, were reduced from 1.00 ± 0.07 to 0.74 ± 0.09 at 0.1 μg/ml and to 0.61 ± 0.05 at 0.2 μg/ml. In contrast, parallel experiments showed that antibody blocking had no impact on IL-1–induced antiapoptotic signals, which at levels of 0.90 ± 0.04 and 0.96 ± 0.05 at 0.1 μg/ml and 0.2 μg/ml of TILRR antibody, respectively, were not statistically different from control cultures (1.04 ± 0.04) (Supplemental Figure 2D).

**Figure 4:**
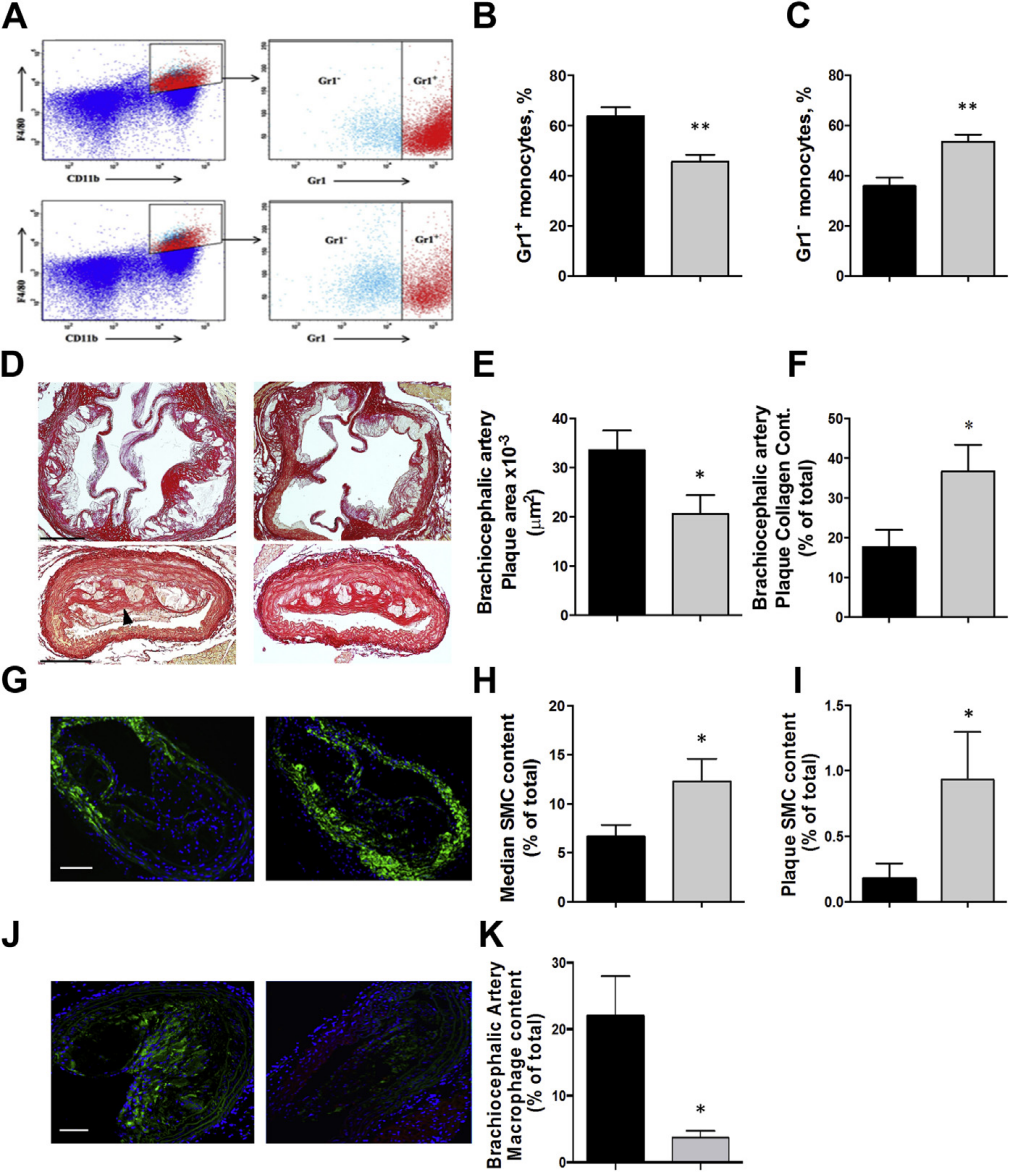
Administration of a Peptide Antibody Targeting the TILRR Functional Site Reduces Development of Atherosclerosis **(A)** Fluorescence-activated cell sorting analysis of GR1^+^ and GR1^–^ blood monocytes as described in the Methods from apolipoprotein E–deficient (ApoE^–/–^) mice on a high-fat diet (control, **top**) and littermates injected with a blocking anti-TILRR peptide antibody 2 times/week for 12 weeks **(bottom)**. **(B, C)** Quantitation of data from experiments such as in **A** shows **(B)** high levels of GR1^+^ monocytes in samples from control animals **(black bar)** and reduced levels in TILRR antibody–injected mice **(light gray bar)** and **(C)** low levels of unactivated GR1^–^ monocytes in control mice **(black bar)** and high levels in samples from TILRR antibody-injected mice **(light gray bar)**, with no change in total levels. Data show **(B)** GR1^+^ and **(C)** GR1^–^ monocytes expressed as percent of total. Values are mean ± SEM. n = 5 control mice **(black bar)**, 5 TILRR antibody–injected mice **(light gray bar)**. Levels in antibody-injected mice versus levels in control mice, **(B)** **p = 0.0065; **(C)** **p = 0.0064. **(D)** Collagen-stained sections of aortic root **(top)** and brachiocephalic artery **(bottom)** from control ApoE^–/–^ mice (**left**, **arrowhead** shows fibrous cap) or TILRR antibody–injected mice **(right)**. Scale bar =200 μm. **(E, F)** Morphometry of **(E)** plaque area and **(F)** and collagen content in the brachiocephalic artery in control **(black bar)** and TILRR antibody–injected **(light gray bar)** mice. Data are expressed as **(E)** plaque area × 10^−3^ and **(F)** percent area of total, and show mean ± SEM. n = 9/6 control mice, 8/6 TILRR antibody–injected mice. Levels in antibody-injected mice versus levels in control mice, **(E)** *p = 0.0434; **(F)** *p = 0.029. **(G)** Sections of brachiocephalic arteries as in panel **D** were stained using an anti–smooth muscle cell (SMC) α-actin antibody **(green)** and nuclear staining (DAPI, blue). Scale bar = 50 μm. **(H, I)** Quantitation of smooth muscle cell content in the **(H)** media and **(I)** plaques in sections as in panel **(G)**. Data show stained area expressed as percent of total and represent mean ± SEM. n = 7 control mice **(black bar)**, n = 5 TILRR antibody–injected mice **(light gray bar)**, for each graph. Levels in antibody-injected mice versus levels in control mice, **(H)** *p = 0.0414; **(I)** *p = 0.0459. **(J)** Sections of brachiocephalic artery from control **(left)** and TILRR antibody treated mice **(right)** stained for Mac3 (macrophage marker, **green**). Scale bar =150 μm. **(K)** Quantitation of macrophage content in sections of the brachiocephalic artery as in **J**. Data show stained area expressed as percent of total and represent mean ± SEM. n = 8 control mice **(black bar)**, 6 TILRR antibody–injected mice **(light gray bar)**. Levels in antibody-injected mice versus levels in control mice, *p = 0.0214. Gr-1 = Myeloid differentiation antigen of the Ly-6 family, (Ly-6G/Ly-6C; TILRR = Toll-like and interleukin 1 receptor regulator.

Repeated injections of the blocking TILRR antibody over 12 weeks had no impact on the level of total monocytes, but caused significant changes in the proportion of monocyte phenotypes. Activated (Gr1^+^) blood monocytes were reduced from 63.78 ± 3.55% in control mice to 45.63 ± 2.81% in antibody-injected mice, whereas the fraction of the unactivated phenotype (Gr1^–^) was increased from 35.87 ± 3.39% to 53.55 ± 2.92% (Figures 4A to 4C).

Histological analysis revealed pronounced reductions in plaque development in the aortic root and the brachiocephalic artery (Figure 4D). Quantitation showed a decrease in the plaque area from 33.53 ± 3.97 × 10^3^ μm^2^ to 20.67 ± 3.74 × 10^3^ μm^2^ in the brachiocephalic artery, corresponding to a reduction close to 40%, and similar to effects observed in the DK mice (Figure 4E, see Figures 3C and 3D). Characterization of the lesions demonstrated an increase in the collagen content from 17.63 ± 4.36% to 36.67 ± 6.30%, and in the number of smooth muscle cells in the media from 6.7 ± 1.2% to 12.3 ± 2.3% and in the plaque area from 0.20 ± 0.10% to 0.93 ± 0.36% (Figures 4F to 4I). In addition, antibody blocking caused a reduction in macrophage content from 22.0 ± 6.0% to 3.3 ± 1.2% of the total area, in agreement with effects observed in the DK mice, and consistent with an increase in plaque stability (Figures 4J and 4K, see Figures 3F and 3G).

### Increased TILRR expression controls inflammatory responses in the lung

**Figure 5:**
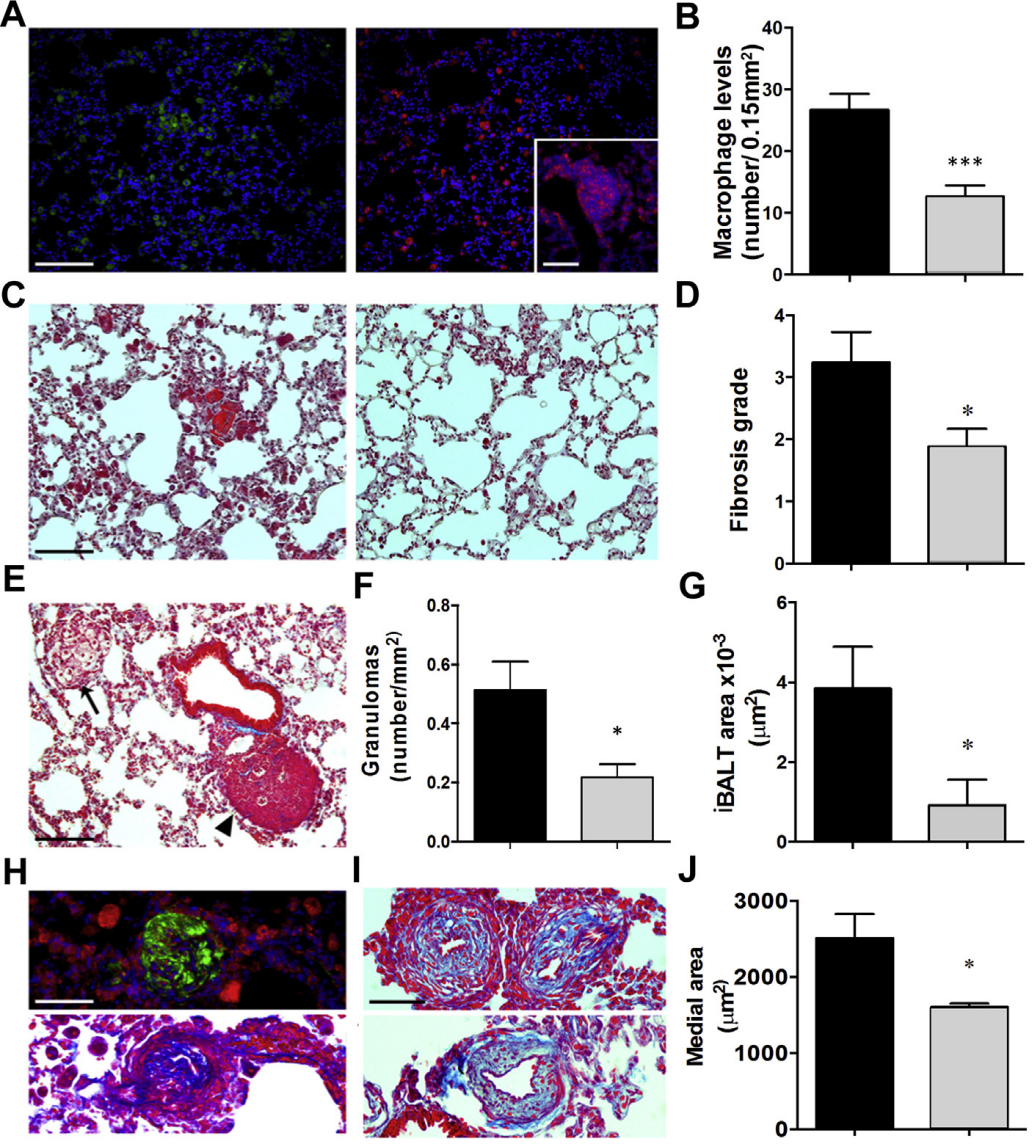
TILRR Antibody Administration Reduces Inflammatory Responses in the Lung **(A)** Macrophages in lung sections from ApoE^–/–^ mice fed a high-fat diet (**left**, Mac-3 **green**) and inducible bronchus-associated lymphoid tissue (iBALT) structures **(inset)** show high TILRR expression **(right, red)**. Scale bar = 150 μm. **(B)** Quantitation of macrophages from images as in **A**. Data are expressed as number of macrophages per 0.15 mm^2^ and show mean ± SEM. n = 9 control mice **(black bar)**, 7 TILRR antibody–injected mice **(light gray bar)**. Levels in antibody-injected mice versus levels in control mice, ***p = 0.001. **(C)** Trichrome staining of lung sections from ApoE^–/–^ mice from control **(left)** and mice injected with the anti-TILRR peptide antibody **(right)**. Scale bar =150 μm. **(D)** Fibrous grade, (Ralf-Harto Hübner scale) in control **(black bar)** and TILRR antibody–injected **(light gray bar)** mice. Data show mean ± SEM, n = 9 control, n = 7 TILRR antibody–injected mice. Levels in antibody-injected mice versus levels in control mice, *p = 0.0472. **(E)** Granulomas **(arrow)** and iBALT **(arrowhead)** in lung tissue from an ApoE^–/–^ mouse. Scale bar =150 μm. **(F, G)** Quantitation of sections as in panel **E** shows **(F)** levels of granulomas and **(G)** iBALT structures in control mice **(black bar)** and TILRR antibody–injected mice **(light gray bar)**. Data are expressed as number of structures per cross-sectional area, as indicated. n 9 control mice, 7 antibody-injected mice. Levels in antibody-injected mice versus levels in control mice, **(F)** *p = 0.0263; **(G)** *p = 0.0452. **(H)** Lung section from a control animal with muscularized vessels with TILRR expression in surrounding tissue (**top**; TILRR, **red**; SMC α-actin, **green**; DAPI, **blue**). Trichrome staining **(bottom)**. Scale bar = 75 μm. **(I)** Muscularized vessels in a control **(top image)** and in a TILRR antibody–treated mouse **(bottom image)**. Scale bar = 75 μm. **(J)** Morphometry of medial area in vessels from control **(black bar)** and antibody-injected mice **(light gray bar)**. Values are mean ± SEM. n = 7 control, n = 7 TILRR antibody–injected mice. Levels in antibody-injected mice versus levels in control mice, *p = 0.0152. Abbreviations as in Figures 1 and 4.

A parallel set of experiments examined the impact of TILRR antibody blocking on levels of lung granulomas and fibrosis in ApoE^–/–^ mice (23). Immunohistochemistry of cross sections of the lung in control mice demonstrated high levels of TILRR expression correlating with infiltrating macrophages (Figure 5A). Staining was particularly prominent within iBALT. Macrophage levels were reduced from 26.67 ± 2.60/0.15 mm^2^ to 12.71 ± 1.77/0.15 mm^2^ following antibody administration (Figure 5B). In addition, antibody targeting reduced the level of lung fibrosis observed in control mice, resulting in a decrease in the fibrotic grade from 3.23 ± 0.50 to 1.89 ± 0.28 (Figures 5C and 5D). Quantitation revealed a 50% reduction in the number of lung granulomas from 0.50 ± 0.10/mm^2^ to 0.21 ± 0.05/mm^2^, and a 75% decrease in the level of iBALT lesions from 3.84 ± 1.05 × 10^3^/μm^2^ to 0.92 ± 0.64 × 10^3^/μm^2^ (Figures 5E to 5G). Immunostaining revealed high levels of TILRR expression in monocytes surrounding muscularized vessels in the lung (Figure 5H). Blocking TILRR function caused a significant reduction in the pronounced vessel thickness in the medial area, characteristic of the condition, from 2,510.0 ± 317.0 μm^2^ to 1,606.0 ± 46.2 μm^2^ (Figures 5I and 5J).

## Discussion

These studies establish a central role for the IL-1RI co-receptor TILRR in disease. The results are consistent with our earlier work, which identified TILRR as a potent regulator of IL-1-induced inflammatory responses 13, 14, 27, 39. We use in vitro and in vivo experiments, which include a recently derived TILRR KO mouse, together with in silico modeling to demonstrate effects on IL-1 receptor function, NF-κB control and gene activity, and to establish the role of TILRR in response to injury and vascular disease. We show that TILRR levels are low in healthy tissue and that expression is markedly increased both in the atherosclerotic plaque and in peripheral blood mononuclear cells from patients with myocardial infarction. The TILRR KO mouse exhibits reduced IL-1 receptor levels, and impaired NF-κB signaling and gene activity, but retains partial IL-1RI function. We demonstrate that genetic deletion of TILRR and antibody targeting of the TILRR functional site reduce development of atherosclerosis, with lesions exhibiting characteristics of stable plaques. In addition, our data show that blocking TILRR function reduces lung fibrosis in a mouse model.

### The Relevance of proteoglycans in signaling receptor function

The potentiating effect by TILRR on ligand-induced activation of the IL-1 signaling receptor is analogous to control of TGF-beta receptor complex by betaglycan (also named, transforming growth factor-beta receptor III, TGFBR3) and of the fibroblast growth factor receptor (FGFR) by Syndecan 4 6, 7. The suggested role of these highly glycosylated co-receptors in enhancing the range and sensitivity of signal control is consistent with their expression in amphibians and higher vertebrates (39).

### Mechanisms underlying TILRR control of IL-1RI

The marked reduction in activity induced by antibody blocking of TILRR function is likely a direct consequence of disruption of the TILRR/IL-1RI complex following blocking of the 448 residue, which is located within the predicted region of protein–protein association 13, 14, 27. A mechanism involving alterations in TILRR binding to the receptor complex is consistent with results from our previous studies, which show that TILRR function is dependent on its interaction with the signaling receptor (13). It is also in agreement with effects of inhibiting co-receptor binding in the Toll-like receptor 4 system, and suggests that changes in receptor conformation contribute to the reduction in signal activation 40, 41, 42. Such alterations are likely to directly impact MyD88 recruitment during activation, a key step in TILRR control of inflammatory responses 13, 14. The potential significance of the 448 site is further highlighted by its recent identification as a single nucleotide polymorphism by the 1000 Genomes Project (International Genome Sample Resource).

### Consequences for TILRR regulation of IL-RI on inflammatory responses

The changes in IL-1 receptor function and the decrease in NF-κB signal activation in the TILRR KO mice, and induced by antibody blocking, are likely responsible for the reductions in monocyte infiltration and activation in the vascular lesions and in the lung. Together with the reduced activity of genes known to be central to development of atherosclerosis and the ensuing decrease in IL-1 production, such changes are likely to have a significant impact on inflammatory response levels and progression of the disease 10, 11, 43, 44.

### The Impact of TILRR and IL-1RI blocking on development of atherosclerosis

The central role of NF-κB and the IL-1 regulatory system in progression of atherosclerosis is well documented, and significant efforts are being made to explore findings from laboratory research in translational projects 45, 46. This study used well-characterized models of atherosclerosis and vascular inflammation, and screening of patient samples to demonstrate a central role for TILRR in fundamental mechanisms of inflammation in vivo, and in development of vascular disease. Future studies, assessing the impact of therapeutic blockade of TILRR on established atherosclerosis, and on mechanisms underlying changes in lipid metabolism and plaque rupture, will include additional in vivo models with characteristics relevant for specific aspects of disease development (47).

Our results show that genetic deletion of TILRR or antibody blocking reduces inflammatory responses and decreases lesion formation during development of atherosclerosis, and thus broadly agree with the impact of blocking the signaling IL-1 receptor, IL-1RI 45, 48. However, in contrast to the vulnerable plaques observed following IL-1RI deletion, characterization of lesions following blocking TILRR function revealed high levels of smooth muscle cells and collagen, characteristics of the stable plaque phenotype (48). The difference in the plaque phenotype may reflect the less pronounced effects on IL-1 receptor function resulting from genetic deletion of TILRR, than reported for IL-1RI KO mice (49). In addition, it may be a consequence of reductions in proinflammatory senescent smooth muscle cells, in combination with maintained antiapoptotic signals (50). The importance of retaining antiapoptotic signals is consistent with our data on TILRR antibody blocking, which showed significant reductions in inflammatory responses but had no impact on caspase activity. Further, it suggests that selective inhibition of inflammatory responses, made possible through targeting the TILRR functional site, may reduce the risk of plaque rupture and related complications 51, 52. In addition, due to its high expression in inflamed areas, blocking TILRR is expected to allow site-specific targeting of dysregulated activation in inflamed areas and reduce the impact on overall tissue function.

## Conclusions

The results are consistent with our earlier work and show that TILRR controls IL-1-induced activation of NF-κB and inflammatory genes and directs signal amplification during host defense responses and vascular disease development. Conclusions are supported by data showing increased TILRR expression within vascular inflammatory lesions, and by the pronounced reductions in aberrant inflammatory activation and disease progression observed by genetic deletion of TILRR and by antibody blocking. Our results highlight the importance of receptor complex composition in disease development and suggest that TILRR may provide an easily accessible therapeutic target for subtle site- and signal-specific inhibition of inflammatory activation during development of vascular disease.

Perspectives **COMPETENCY IN MEDICAL KNOWLEDGE:** The cytokine IL-1 and its signaling receptor IL-1RI are central regulators of vascular disease. Genetic deletion of IL-1RI and antibody blocking of IL-1 responses in animal models reduce development of atherosclerosis but result in development of vulnerable plaques, in part through increased smooth muscle cell apoptosis. Our findings demonstrate that using a more moderate approach, targeting the co-receptor TILRR, a cell surface proteoglycan that associates with the signaling receptor IL1-RI to amplify IL-1-induced responses, allows site- and signal-specific blocking of inflammatory responses, resulting in reductions of plaque development and increased plaque stability.

**TRANSLATIONAL OUTLOOK:** Antibody blocking of TILRR function reduces development of atherosclerosis in animal models to the same degree as obtained by blocking IL-1RI expression and function, but does not induce the side effects observed after targeting the signaling receptor. Development of specific antibodies for distinct blocking of TILRR-induced signal amplification could provide a mechanism for selective targeting of inflammatory responses in a range of conditions. Future preclinical and clinical trials may demonstrate translational prospects of an anti-TILRR therapy in treatment of cardiovascular disease.
