# Supplementary material for: The IL-1RI Co-Receptor TILRR (FREM1 Isoform 2) Controls Aberrant Inflammatory Responses and Development of Vascular Disease
Source: JACC Basic Transl Sci. 2017 Aug 28;2(4):398–414. doi: 10.1016/j.jacbts.2017.03.014 (PMC5582195; doi:10.1016/j.jacbts.2017.03.014)

SUPPLEMENTAL FIGURE LEGENDS

Supplemental Figure 1. Gene targeting strategy for the TILRR KO mice.

TILRR sequence of the floxed allele, was created by inserting flox sites in exons 24 and 25 (15,16). Excision of the floxed sequence by Cre was carried out to eliminate expression of the functional TILRR product from the null allele.

```
TTGCAGTGGAAACAAAATGTTGAAACATTATCAAAAACCAGTGTTACTGGGTGACTGGCTG
CAGTGAATGGAATAGCCCTAGTTTTTGACGTAAGGAAAACATATACTAAAGATTGTAAGAG
AGGTCTACTGACCCCAAACTTGTGTCATTTCTACTTTCTCTTCAAAGTTCGACATTGGTAC
TTGGGGACTGCAACTCTTGATTTTTTTGTGTGTAGGGGGGATAGAGGGGTTAAGCTATAGA
TTGAAAAGTCTGTAAGAAGAGAGGATGCAGAGACAAATAATCTGAGTTCTGGTACTAACTC
TTATCTACATGTTCATGCCCTCTACTAGGTTTCTAAATTTCTCTTTTCAGCAGGGAAAGAT
CCTTGTCCATCATCAATACCTACATATCTCAAATGAAGCAGGTGCAGAAGAACACATGTAG
GCCCTAAGAGGTTAGAGTCTCACATAATACCGcggagctgggtggcatctagtattcatct
ggcctatcctctcatcattttacaaatttaggataagaagtcccagaaaggagagtgagct
agataagttaacatgactaaataatgTGATCTGCCCTTTACAAATGTTCTTACAAACACTG
                   EcoRI (110746)                                loxP
CTGCCAGACACAGAATTCCTGCAGCCCAATTCCGATCATATTCAATAACCCTTAATATAAC

                                                          lox 3F
                                            XhoI   BamHI (110838)
TTCGTATAATGTATGCTATACGAAGTTATTAGGTCCCTCGAGGGGATCCCACATCTGCAAT
CTCAGCGTTCCCGTGGTGGCATGGGAGCTGGTGACACAAGAATCTCTGCAATCTCGGGGGT
GGGTGTGGGGGCACAGCTAGCCTGTCGTATACAGCTGCAAACAACAGAACTTGTTTCAAAT
AAGGTGGAAGGTAAGACCTGACACTGATAGGCAGATTGTCCAATGACTCTGCACATGTGCT
GTGGCAAGCATACACACTCTCACAACCCCTCTCCCacagagggagacagagacagagagac
agagagacaaagagacacagagacagagagacagagagacagagagacagatagaAACGGA
AttttttttCAGAAATAAAAAGGATGTTTGAGAcagtggtagagccctagactagcacgtg
taaagctctgggttcaatacccggtactgagaaaagcatgaaaatatacaaattagactaa
atTATCCTATTCAGTGTCTCATTATACCATGGTCTTTAAAGTTCTGTTTTCTACATTCCAT
GAAAAAATATTACTGCAATATTAAAATATATGAGCACACAGTAGCTACCCGTACAATGGGT
CCAGTGCGACAGGAAATTCTTGCTTGGGACTAAAGATTACACTGAGTTTGCCTGCTTCATC
GTAGGAGGGGTATCAGTGATGCTAGGAAAATACTCTTGGATGTAGTATGACCAATGTGTAT
GTTGCTTAGCAACTGGTTTACTAACAGAAGGATGCTCCTCAGGAATCCCCTCCCTGCTTTC
CCCTTTGAGTCTCAATGTGGTGTGTCCTGTAGTAAAGGTGTGTGTGTTATCATTTCATTCC
     Translational start of TILRR->                          Exon 24 of
AGTCGATGGGGACACAAGAGCCCATGCTGAAGGCTGCCTTGCCTCTCTTTGCCAGATTTAC
   ▸ M  G  T  Q  E  P  M  L  K  A  A  L  P  L  F  A  R  F  T
                                                         ▸ F  T
CATCAGCAATGGACTGCAGACCCAGCGTGGGGTGTTTGAAATCACACTGCAGACTGTGGAC
▸  I  S  N  G  L  Q  T  Q  R  G  V  F  E  I  T  L  Q  T  V  D
▸  I  S  N  G  L  Q  T  Q  R  G  V  F  E  I  T  L  Q  T  V  D
AGCGCCTTGCCTGTGCTGACCAAGAACAAAAGGCTGAGGCTAGCCGAAGGGGCCATGGGCC
▸ S  A  L  P  V  L  T  K  N  K  R  L  R  L  A  E  G  A  M  G
▸ S  A  L  P  V  L  T  K  N  K  R  L  R  L  A  E  G  A  M  G
TCCTGTCCGCTGATCACCTTCAGCTGACTGACCCCGACACACCTCCAGAGAACCTGACCTT
▸L  L  S  A  D  H  L  Q  L  T  D  P  D  T  P  P  E  N  L  T  F
▸L  L  S  A  D  H  L  Q  L  T  D  P  D  T  P  P  E  N  L  T  F
CTTTCTGGCTCAGCTCCCACGCCACGGGTACCTCTTCCTGAGAGGGAAAGCACTCCAACAC
▸  F  L  A  Q  L  P  R  H  G  Y  L  F  L  R  G  K  A  L  Q  H
▸  F  L  A  Q  L  P  R  H  G  Y  L  F  L  R  G  K  A  L  Q  H
AACTTCACCCAGCGAGATGTGGACAGCGGGGGCGTGGCCTACCAGCACTCAGGAGGCGGCG
▸ N  F  T  Q  R  D  V  D  S  G  G  V  A  Y  Q  H  S  G  G  G
▸ N  F  T  Q  R  D  V  D  S  G  G  V  A  Y  Q  H  S  G  G  G
CCCGGGAGGACTATTTTACTTTTCTAGCCACAGACAGGAAGAACCAAGGCTTTGTTGTGGA
▸A  R  E  D  Y  F  T  F  L  A  T  D  R  K  N  Q  G  F  V  V  D
▸A  R  E  D  Y  F  T  F  L  A  T  D  R  K  N  Q  G  F  V  V  D
TGGGAAAGTTCAGAAAGAGCCTGTTCGCTTCACAATCCAGGCAAGTATGGCAGGCACGTag
▸  G  K  V  Q  K  E  P  V  R  F  T  I  Q
▸  G  K  V  Q  K  E  P  V  R  F  T  I  Q
tttgtgccctcatggtggaaagcatggtagctggcaggcatatcctgaagcagtagctgag
agcttacatcctgacccacaagcccatggctgagagagtaagacagggtctggcatagacg
tttgaaacctcaaagcccacagtgccaacaagcacacaccatctaatctttcctaagcagt
tcaccaactggggtctaagtgttccaatacatgagctatggggcctatcccattcaaacta
ccaTGCCTCCTCATATTTGAAAAGCATGCCTGCAGAATGTGGTGGCAATAAAATAAGATTA
GGTTATCCAAGGCACTCACACATTGCCTGAAACGTATTTGAGGTATGTATTAGACTTTTGA
```

AATTATAATTGTGCCTTTAGGATTCTGCCTAGTCAGGCAGGTCGTTTACTGGCATTGTAAG
TATAGAAACTTACAAGGATCCATCAGTATCACCCAGGAGCTGATGATGGCGATAACAGTAG
CAGCAGCTACAAACACCCTTTTCTCACTTTTAATTCTTAGATAGTTCCATGGACCTATATC
ATTCTCATTTTCCCATTCCCCCACTCATCCCCCTACCCTACCCATTGAAACTATTCTCCAA
CATCTtgtgtgtgtatgtgtgtgtgtgtgtgtgtgtgtgtgtgtgtgtgtgagtgtg
tgCGCGCGCGCGTGtgacccactgagtttgattagtgttgctcccatgagcgtgggtagat
tattcgctggagcacgaggaacatctcaacagcgacatcactgaagaaaacaatgctctct
cctccagcaaccattagccaataggacttcagggggcagtggtgaagctttgtggtccctc
ccatacccatgatgaaatgtgggtggaccccgtcttgtgcaaatcatcagcggagcagcat
ccttgagtgctatggccttgtcatatctacatgtcgtttttacagtccatctccccatcct
ctggctcttccatgctctgcATGCTTTTAAAATGGTTATCTAAAGAGCAGGGCTACTTTGC
ATAAatctcatttgattctcataaacattgtatgtgctaggttttattgtctacatttcta
tacacgaagaagctgagtatcagaagttaaataatttgatcaaagACGTCAAGTCTGTTTG
ATTCCAAATTCTGTGTCCCTCCTATGGCTCCATACGGCCACATTTGTAGACTCTCAACAAT
TCTTGGAGGGCAGAGTGTTTTTCGTCTTTTAGTCAAAACACATCAAGTATGTTCTGGCTCT
CAAAACTCTTACACGTACTCTTCTTGGATGGGGTTCACACTGCTCCACACACTTCAACCAA
CAGCAACAAAGATGGGATTTTGTCTCTACTTCAGGGTACCCTCTGACTAGTGGGATACTAT
TTTCTCCTTGTTTTTTAGCCTCTGTGTACAGTATCCATATATCTGTGTAGATACACAATAC
AGATCAATAGGATGTGAGGGACAAAGTGTGTGTCTGAATGACAGAGAGGCACACAGGCACA
AGTTGATTTAGGAATTGCCTTATGCTCAAGATGTATTGTGCAAGCAGGCCAGTAGACTAAA
ACTGGCAGTCAAGGTGATATGGTGGCCTTGGATCTAAAAAGGGTAGAGAGACAAAGCTCTT
ACTTCTTTGGGGGAATCTCGGTCTTCTCTCTTTGGACTGGCAGTGGATAATTCTCACTCAG
TCTACATTACCCAGTCAATCCAGGCCTATGGCCTAGAAGACTTGTCACAGAATTAACCTTC
AGGGTCTCTGGGTGTGGATGAGTCTTTGGCACTATGAAGACTGTAGATACTTGGGGAGATG
GTGTTGATGTCATCAATACACAGGCACTATGGTGTCTACTGAAAAATGTTTTTCTGGATTT
GTTTCCTGAAAGTTTTGAAGAGTTACAAATTCTATATGCAGACACAACTGCTTTCTGTATT
CCAGAGATGGAAATGGGGGAATAGGGAGAATGTGCTAGAACTAAAACAGATCTCTCCACCA
GAGCGTTCCTCTAACTGTAAAGCTAGTCTAAGAGTTTGTTCCCTGTCTGAAGTTGTGGCAG
CTCTCTGTTGGGTTTGGTAAAGCTTATTTTTCATCACAAATGTAAGATGTCCCTTGTGCaa
acaaacaaacaaaaaaaacTTGTGTTAAACACTTACGGGGAAAGCATGTGGTTTTTGTCTT
CAAATTTCCTTGACCCTTGGTCCCTCAGAGCAGAGAGGACAAGGAGGGTTTTATGAAGCAG
CTGGCCCTGCCAGCAATGGAGAAGAGAAAACATGCCCAGTGTCTGGCTTCCAAGAGTTGTT
TTGTGAAGACAATACAGTACTTACTGACTGCCACCAAATGATACACTGACCCAGATAAGTA
ACTCAAGCATGGATCAACAGGTTCTGTGTGGTTCTAAGAGTAACTATGAGTAACCAAGGGA
AAAGCTGCTGGATGGAAGAATTAAAAATAACagatagacagatggatggatggatggatag
atagatagatagatagatagatagatagatagatagatagacagacagatagataTGGTGT
GGCTGACTAATAGCAGGAGGCACACATGACCTTCATTCTGTGGGAAACCACACATGCTCAA
GACTGTCACAATCTATAGTCCTCCTGTGAAGGGGACAAGGAGTGTATGTAGACAAAAGGAC
CTGCCATTACTCTCTATGGGGACTGACATGTCTGGGAAGAGTCTTACTAAATATACCTTAA
GTTATCGTAAAATACGCAGAGCCTTCCTTAAAAATAAAATGTCTTCCCCTGTAATGTTTCA
AAGCCTTCTGGTCTACCAAGTCAAAGACCTGCTTTTGATGATGCAAAATGTTTAATATATA
TGAACAAACCAGTATGGCCAGAGGCACAACAGGGGAACCAAAGTGCCCAGCGCTTGTGTAG
CAAGACATTTTTAAAAACATTGACCACAACAAATCTTTATTGCTTTTGTAACTTTTGGGTT
TATAAACATAACAACAAAATAAAAGCCACAACCCCACCTTCAGCCACCTGGGTTTGGGAGG
ATCCTAATATGGCTTAGTATGTTTCATGTTTCCTCTTTCATGCCACCGTGACTACGGGAGC
TGCTCTTCCCAGGAGATCATCACGAAATTCACAGCCCGAATTCCACCAGCCTTGAAGTGCT
GAAAGTCTCAGCATACAATTTTCAGATCCGAATTCGTTAAAATGCTTTTATAGTCATCTAC
AACAAATCCAGAAAACATAAACACGCttttttttGCATGCATATGCGTGTAGGGTGTGTGT
GCATGCTTATTTACAAATGTGAGAGCATGTTTGTGTGCTCAtgtgtgtatgtatgtccatc
tggtggctagagggtgactttgagtgtcttccttgattactctattttccaatggcaaggg
ctcccttgttgaacacggagctagctagttcagctaatctggctagccagcttgccccagg
gatatcatctctgacacagccttacccaatCCACCACCCTTAAGTATCTAGTCCACTTATT
GAGTCTGGTGGTCTTCAGCCTTAGTGGAACAATATACAGGCTAAAGATAATCACTTGCATA
ATTTTTATAGTGAACTATTTCAGCTTTTCTTCTCAACACTTTGGCATTTGGGCAAAGGCAT
GTGGGAAAGGTCTTTGGCATTTGTAAAAACCTAGGATTCTGTTTATGTAGTTCATTAAACA
CAGCAAATCTTTCCGTGGTGCTGACGTTAAATTTGCCATGTAATTTTGGGGTCATTTTTAG
TGTTTTCCTTGTGCTTCTGTCATGGTTCATGCATAGTGCTGTGTGCAAGAATGGGCATTGG
GTGGGAAGGTGTGGTATGTCCCTTCATAAAGCTAATATTGTAAGGAAATTCAGACAGGGGA
CATCATAAAGCTGGGTGGAGGTTTGTGATGGAAAGGAAACCTTGGCCCTTGTCTCTGTATT
TGTTAGGATAGGGTGAACAAGACAGGCTTCGGCAGAAATCAACAGATAAGGACTCCAAGCG
ATCTCTGGGTTGAGGAGTTCACTATTGTTGAGCCAGGAAAGCCCTGTGTAGCATAGGGAGA

Exon 25 of Frem1
Exon 2 of TILRR

GAAGTCCTTGTGTAACGCTACGTTGGTGATTCAGGTGGATCAGCTGGACAAGGCAGCGCCC
▸ V D O L D K A A P

CGTATCACACACTTGCACTCCCCTACTCAAGTGGGGCTCTTGAAAAATGGCTGCTATGGGA
R I T H L H S P T Q V G L L K N G C Y G
TTTACATCACTTCCCGTGTGCTGAAGGCATCAGACCCTGACACAGAGGATGACCAGATCAT
I Y I T S R V L K A S D P D T E D D Q I I
CTTTAAGATTTTACGAGGCCCATTGTACGGACGTCTGGAGAACACAACAACAGGTACTTTC
F K I L R G P L Y G R L E N T T T

Frt (1
EcoRI (116521)
CATCTTTTTTGGGGGGGCGGGTGTACTTTCCATCTCAGTCCTTGTAAGTCCTCGAATTCCG

XbaI (116541) loxP
AAGTTCCTATTCTCTAGAAAGTATAGGAACTTCATCAGTCAGGTACATAATATAACTTCGT

BamHI (116619)
ATAATGTATGCTATACGAAGTTATTAGGTGGATCCGTTGCTGGCAGAGCTAAAGAGGAACT
CAGGTAAGTGTAGGTAGGCCCGTTTGGTGAGTGCTCCCTAGGGCTTGCTGATAGACTCAGT
TTATAAATCAATGTTGTTGCTAATAGGGGTTTGAAGCTGAATCTTCTAAATTCATCTTCAT
AAGGAGAAACAGCTCCTGACACACAGTAGCTGAACTCTTCACTGACTTGTTATTTTGTTTT
ATTTTCATGTTGTGTTTTATTCAGAACACAGTGCCAGGGTGAGATTGATATTTGAGATTAA
TTTCTTTCTCCTAGTATGAATCCCCAATTATATCATCTGCTTTTTTAAAAATATGGTTTTT
CCCCTAATAGCTTTGCTAAATTTTATATTAGCTCTGCTTCAGGAAGGCAATGATCACAACA
ACTCATTAAACAGGATGGCAGAGGCGACAGGTTTTATTAACTCCCCATTCCCTTTCTCTTT
AGTTTAGGATTATAAGGCAAGTATCTCCAAAGCAAAGCAAACTTAAAGAGGAATTTGAGTC
AATCTTATTTGTATCTGAGCATGGGTGCAACAATGCTAATTTTCTTTAAATGGACTTTGGC
ttttttttctctctctttaaaacaaggtctcaggtagcggctggcctcaaatttgatatgt
agtagaacacagcc

Supplemental Figure 2. The blocking peptide anti-TILRR antibody, targeting the functional TILRR residue D448, selectively inhibits NF-κB induced inflammatory responses.

a. Space-filled representation of the predicted TILRR structure and TILRR/IL-1RI binding interactions. The tertiary structure of TILRR (NP_001171175.1), obtained using a *de-novo* approach in I-Tasser Zhang Server, gave a confidence score of -2.14. IL-1RI was generated using the resolved crystal structure (PDB: 4DEP) and binding interactions by Gramm-X, and modified as described in the Material and Methods (33-38). The model predicts TILRR binding to the convex region of the extracellular domain of IL-1RI. IL-1RI (red); TILRR (blue).

b. The predicted location of the functional TILRR site at residue D448 is within the binding region of the two proteins (13,14). Rotation 45 degrees in relation to A; TILRR (blue); D448 functional site (red); IL-1RI backbone (silver).

c. Hela cells were pre-incubated with an anti-TILRR peptide antibody, targeting the D448 residue and stimulated with IL-1 (10^{-9}M, 6hr). Increasing the antibody concentrations results in a successive reduction in IL-8 gene activity. Data show levels of activity in the presence of the blocking TILRR antibody relative to activity induced in the presence of the equivalent level of a non-specific IgG. Mean±SEM, n=4, *=p<0.05, **=p<0.01.

d. Cells were pre-incubated with an anti-TILRR peptide antibody targeting the D448 residue and stimulated with IL-1 (10^{-9}M, 6hr), and caspase activity determined as described in the Material and Methods. Data show levels of activity in the presence of the blocking TILRR antibody and are expressed relative to activity induced in the presence of a non-specific IgG. Mean±SEM, n=3, ns.

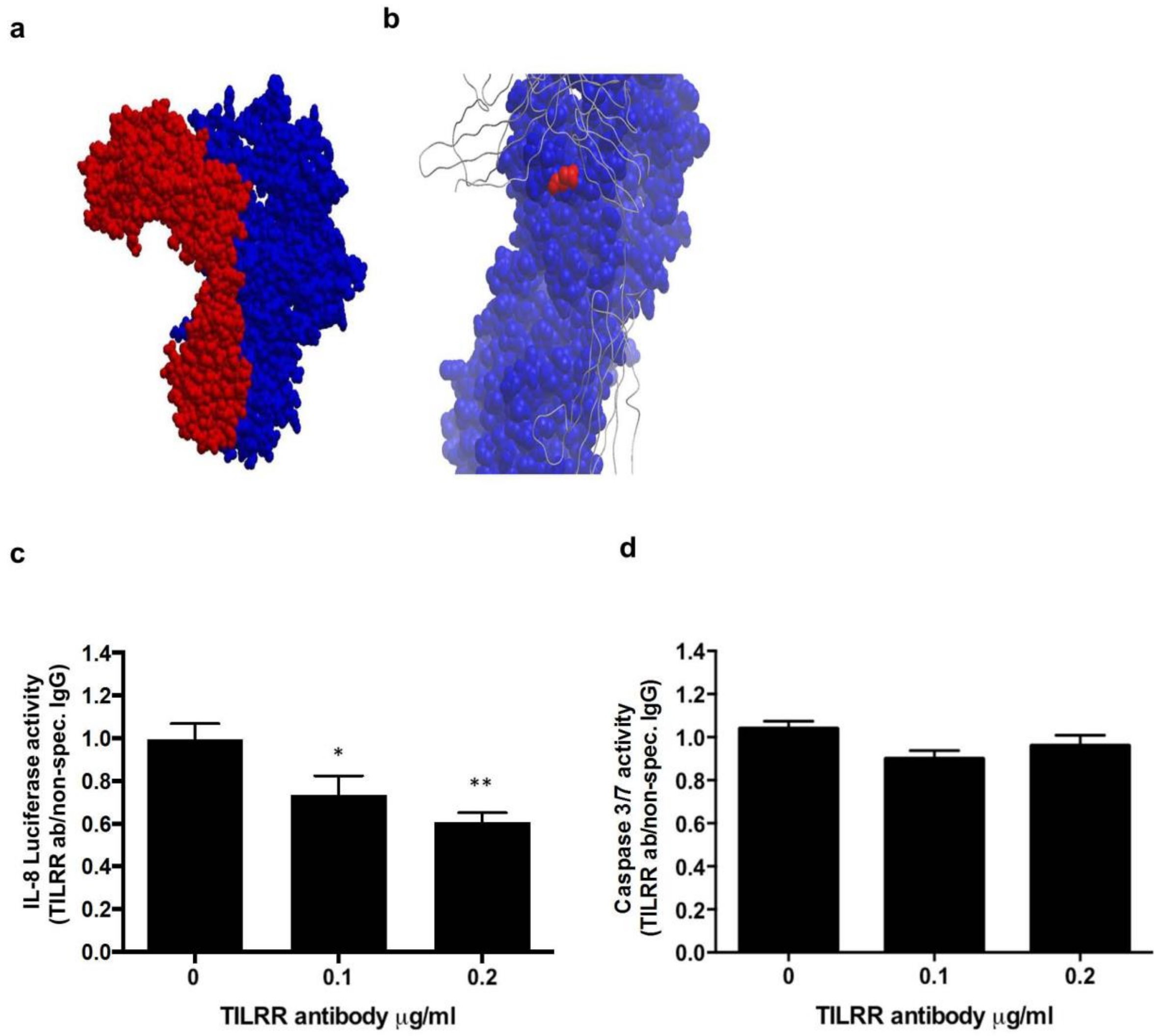
a
b
c
d
1.4
1.2
1.0
0.8
0.6
0.4
0.2
0.0
IL-8 Luciferase activity
(TILRR ab/non-spec. IgG)
*
**
0
0.1
0.2
TILRR antibody μg/ml
Caspase 3/7 activity
(TILRR ab/non-spec. IgG)

Supplemental Figure 3. Computational modeling predicts TILRR knockout to affect gene activity.

a. Simulations using agent based modeling, of IκBα levels during IL-1β stimulation in TILRR KO and wild type cells (27-32), predict a significant reduction in inhibitor degradation in TILRR KO mice, in agreement with biological data shown in Figure 1G. Mean±SEM < 2%, n=3, ****=p < 0.0001 at 60 min.

b. Model simulations predict a pronounced reduction in IL-1β-induced inflammatory gene activity in TILRR$^{-/-}$ cells relative to activities in wild type cells. Mean±SEM, n=3, **=p< 0.01.

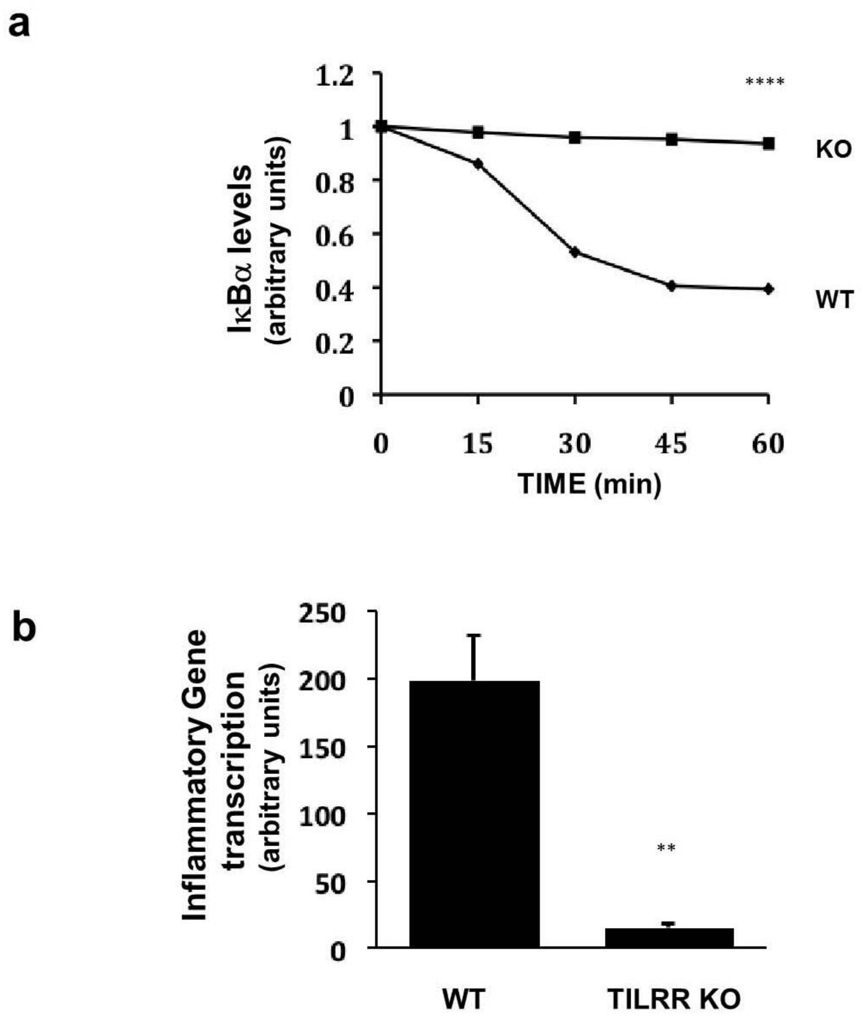

Supplemental Figure 4. TILRR knockout reduces activation of inflammatory genes.

Microarray analysis demonstrates pronounced reductions in gene activity in blood and spleen from TILRR knockout mice during an inflammatory response. Expression profiles in wild type and TILRR KO mice were analyzed using R Bioconductor 2.14 (http://bioconductor.org/) and DAVID 6.7 (https://david.ncifcrf.gov/) (25,26), and data expressed as log2 fold change (Log 2FC) of activity in TILRR KO relative to activity in wild type for each gene, n= 3 independent experiments.

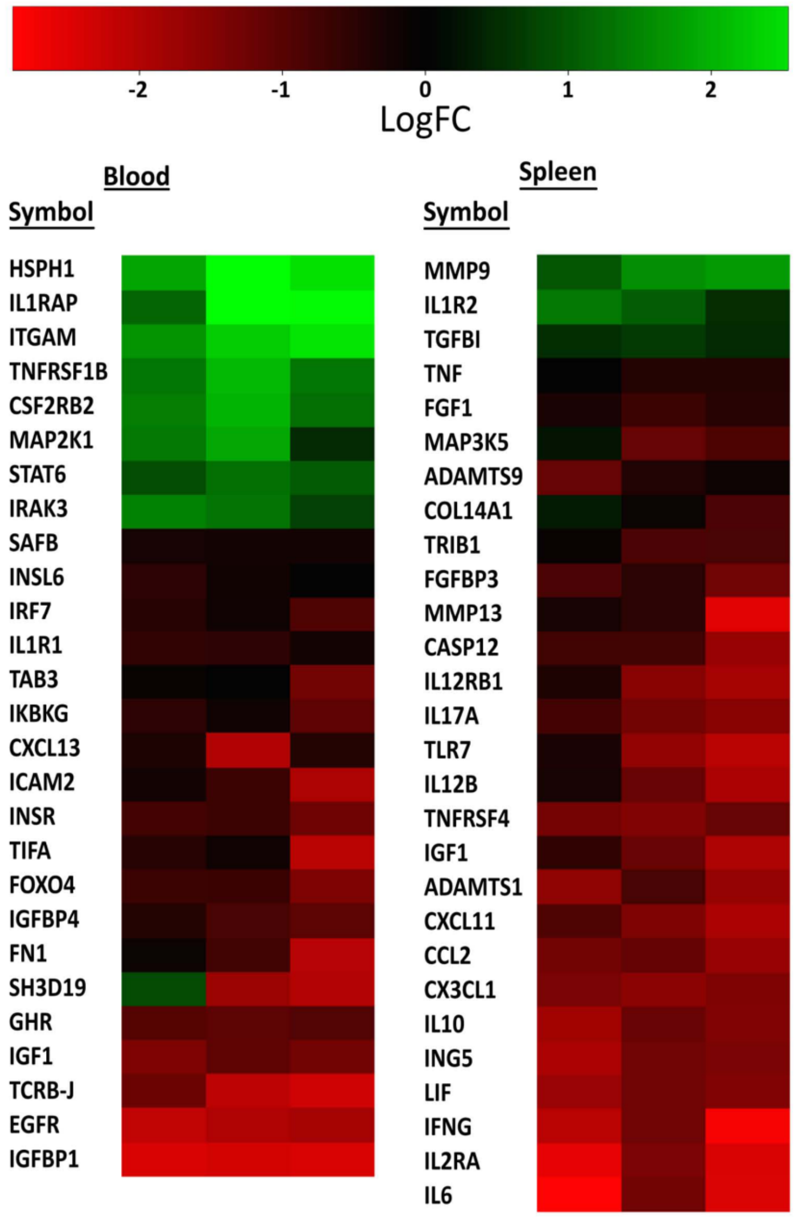

Supplement: Supplemental Figures 1–4 [file mmc1.pdf]
